# PACAP/GCGa Is an Important Modulator of the Amphioxus CNS-Hatschek’s Pit Axis, the Homolog of the Vertebrate Hypothalamic-Pituitary Axis in the Basal Chordates

**DOI:** 10.3389/fendo.2022.850040

**Published:** 2022-04-14

**Authors:** Jason S. W. On, Liuru Su, Hong Shen, Aloysius W. R. Arokiaraj, João C. R. Cardoso, Guang Li, Billy K. C. Chow

**Affiliations:** ^1^School of Biological Sciences, The University of Hong Kong, Hong Kong SAR, China; ^2^State Key Laboratory of Cellular Stress Biology, School of Life Sciences, Xiamen University, Xiamen, China; ^3^Comparative Endocrinology and Integrative Biology, Centre of Marine Sciences, Universidade do Algarve, Faro, Portugal

**Keywords:** CNS-Hatschek’s pit axis, hypothalamic-pituitary axis, amphioxus, PACAP/GCG, GHl

## Abstract

The Hatschek’s pit in the cephalochordate amphioxus, an invertebrate deuterostome basal to chordates is suggested to be the functional homolog structure of the vertebrate adenohypophysis based on anatomy and expression of homologous neuroendocrine genes. However, the endocrine potential of the cephalochordate Hatschek’s pit remains to be demonstrated as well as the physiological actions of the secreted neuropeptides. In this study, we have explored the distribution and characterize the potential function of the amphioxus PACAP/GCG precursor, which is the ortholog of the hypothalamic PACAP neuropeptide in vertebrates. In amphioxi, two PACAP/GCG transcripts *PACAP/GCGa* and *PACAP/GCGbc* that are alternative isoforms of a single gene with different peptide coding potentials were isolated. Immunofluorescence staining detected their expression around the nucleus of Rohde, supporting that this structure may be homologous of the neurosecretory cells of the vertebrate hypothalamus where abundant PACAP is found. *PACAP/GCGa* was also detected in the infundibulum‐like downgrowth approaching the Hatschek’s pit, indicating diffusion of *PACAP/GCGa* from the CNS to the pit *via* the infundibulum‐like downgrowth. Under a high salinity challenge, *PACAP/GCGa* was upregulated in amphioxi head and *PACAP/GCGa* treatment increased expression of GHl in Hatschek’s pit in a dose-dependent manner, suggesting that *PACAP/GCGa* may be involved in the regulation of *GHl via* hypothalamic-pituitary (HP)-like axis similar as in the vertebrates. Our results support that the amphioxus Hatschek’s pit is likely to be the functional homolog of pituitary gland in vertebrates.

## Introduction

The seminal occurrence of hypothalamic-pituitary (HP) axis was an important innovation acquired during the early evolution of vertebrates, and this enabled the regulation of many complex neuroendocrine functions such as reproduction, metabolism, growth, and maintenance of internal homeostasis (1). This axis is a central regulatory system for the production, secretion, and action of several peptides and proteins and their receptors which are suggested to have arouse or expanded during the whole genome duplications that occurred prior to the vertebrate radiation (2–6). Invertebrates lack a hypothalamic-pituitary axis, but in the basal invertebrate deuterostome, the cephalochordate amphioxus which is evolutionarily close to the chordates, primitive structures that are likely to be precursors of vertebrate complex endocrine organs such as brain, pineal organ, pituitary, endostyle/thyroid, gut, ovary, and testis have been described (7). Searches in the amphioxus genome revealed that sequence orthologs of various vertebrate hypothalamic neuropeptides and several pituitary hormones are present such as the gonadotropin-releasing hormone (GnRH) (5), kisspeptin (8), vasotocin (3), and thyrostimulin (3), the most-like ancestral glycoprotein hormone percursor that originated the vertebrate gonadotropins [follicle stimulating hormone (FSH), luteinizing hormone (LH), and thyroid stimulating hormone (TSH)] (3), suggesting that amphioxus might possess the foundations of the vertebrate neuroendocrine system (7, 9).

The nerve cord-Hatschek’s pit is a ventral lobe of the amphioxus brain that ends near the dorsal surface of a Rathke’s pouch-like structure and is suggested to be comparable in function and structure with the vertebrate hypothalamic-hypophyseal neurosecretory system (10). Despite the detection of putative homologs of the vertebrate anterior pituitary-regulating factors, the physiological role of the nerve cord-Hatschek’s pit connecting the region currently remains poorly understood. The understanding of the peptidergic potential of the Hatschek’s pit has been controversial, and *in situ* hybridization failed to detect the expression of the two thyrostimulin subunits (*gpa2* and *gbp5*) and *vasotocin*, and this was taken to suggest that amphioxus lacked a pituitary-like structure as an amplifier of the endocrine signal (3). However, expression of endogenous growth hormone-like peptide was found (11) and therefore, the resemblance of Hatschek’s pit to vertebrate hypothalamic-pituitary axis remains to be further clarified.

Recently, peptides that have shared common ancestral origin with the vertebrate hypothalamic-pituitary adenylate cyclase-activating polypeptide/glucagon-like peptide (PACAP/GCGs) superfamily (aka as the secretin peptide family) which emerged and expanded during the two rounds of whole genome duplication were described in amphioxus (12, 13). In the cephalochordate *Branchiostoma floridae*, a single PACAP/GCG gene that encodes for 3 mature peptides (PACAP/GCGa, GCGb, and GCGc) that are similar in sequence was described, but they share only marginal sequence similarity for the vertebrate orthologs; however, key amino acid residues that are important for receptor function are present (14–16). In vertebrates, PACAP/GCG peptides activate receptors of class B1 of the G-protein coupled receptor (GPCR, aka Secretin GPCRs) superfamily, and in *B. floridae*, a functional PACAP/GCG receptor (*PACAP/GCG-R*) has been characterized (12). In other amphioxi species, homologs exist, but their physiological role remains to be established (13). In other invertebrates such as tunicates (urochordate) and few protostomes (arthropods, mollusc, and planarian), putative peptides that share higher sequence similarity for the vertebrate PACAP peptide have been described, but their existence remains yet to be confirmed (13, 17, 18).

PACAP is one of the most extensively studied neuropeptides in vertebrates. It has been characterized in representatives of most of the major vertebrate phyla, and peptide functions are diverse and include the regulation of neurotransmission as a neurotransmitter, neuromodulator, and neurotrophic factor (19–21). In mammals, PACAP is highly abundant in the hypothalamus from which it is secreted to the pituitary gland and is also found in other brain regions (22–25). In this study, we aimed to understand the role of the cephalochordate PACAP/GCG system (peptide ;precursor and receptor) and mapped its distribution and characterize its potential functions in the brain of amphioxus *Branchiostoma belcheri*. We found that two *PACAP/GCG* transcripts exist and that they have different coding potentials and different tissue distributions. *PACAP/GCGa* was the most expressed transcript in the Hatschek’s pit and an increase in seawater salinity modulated *PACAP/GCGa* gene expression, and this was correlated with the upregulation of *GH-like* transcript (a functional homolog of the vertebrate GH) in Hatschek’s pit in a manner resembling the mechanisms of regulation of GH secretion by PACAP in the vertebrates (26).

## Materials and Methods

### Animal Models and Experimental Conditions

Two phylogenetically related amphioxi species *B. belcheri* and *B. floridae* were used. Sexually mature *B. belcheri* (between 1.5 and 3.8 cm) were purchased from a research center in Longgang marine organism industrial park in Shenzhen, and short-term rearing (<3 months) was performed in aerated tanks filled with artificial sea water (ASW) and salinity was maintained at 25 ppt. Adult amphioxi were fed with the microalgae *Isochrysis galbana* (1 × 10^4^ cells/ml) three times per day (Red Sea Salt, Red Sea Fish Pharm Ltd., Israel) and were used for gene cloning, *in situ* hybridization, experimental salinity challenges, and immunofluorescence staining. The amphioxus *B. floridae* was used for RNA extractions to isolate the full-length *PACAP/GCG* precursors and for whole-mount *in situ* hybridization expression analysis on embryos. This species was obtained from Dr. Li Guang’s lab in Xiamen University (China), and animals were maintained as previously described (27, 28).

### RNA Extractions and Isolation of PACAP/GCG Precursors

Total RNA from whole sexually mature amphioxus or excised adult amphioxus tissues was isolated using the TriPure reagent (Invitrogen, Carlsbad, CA). Four micrograms of total RNA and anchored‐oligo (dT)18 primer were used together with Transcription First‐Strand cDNA Synthesis Kit (Roche) to generate first‐strand cDNA for molecular cloning or quantitative real‐time PCR analysis. The nucleotide sequence of the *B. floridae* PACAP/GCG precursor (Transcript ID: 96553) was retrieved from online genomic database of *B. floridae* (https://mycocosm.jgi.doe.gov/Brafl1/Brafl1.home.html) by tblastn with mature peptide previously reported (9) as query. Specific primers for *B. belcheri* were designed based on the predicted *B. floridae* PACAP/GCG precursor sequence (**Supplementary Table S1**), and PCR reactions were carried out on a C1000TM Thermal Cycler (BioRad, CA). PCR reactions were performed with Platinum^®^ PCR SuperMix (Invitrogen), target cDNA, and 10 pmol of the specific primers. Analysis of PCR products was performed on 1% TAE agarose gel electrophoresis, using SYBR^®^ Safe DNA gel stain (Invitrogen). PCR products of the expected size were cloned into the pGEM^®^‐T Easy Vector and sequenced to confirm identity. After confirmation, specific primers were designed for RACE to obtain the missing 5’ and 3’ region and obtain the complete *B. belcheri* PACAP/GCGs precursor (**Supplementary Table S1**). The 5’ and 3’ RACE reactions were performed using the Platinum^®^ PCR SuperMix (Invitrogen) according to the manufacturer’s instructions. The full‐length *B. belcheri* PACAP/GCG sequences were amplified and subcloned into pGEM^®^‐T Easy Vector and sequenced. For the isolation of *B. floridae*, specific primers were design (**Supplementary Table S1**) for PCR and the amplified DNA fragments were ligated into pGEMT vector and sequenced to confirm identity.

### Quantitative Real-Time PCR Amplification

Quantitative real-time PCR (qRT-PCR) was used to measure the expression levels of the genes with the SYBR green PCR kit (Vazyme, China). The reactions were performed on the StepOnePlus™ Real-time PCR System (Applied Biosystems), and data were analyzed using the StepOne Software v2.1 (Applied Biosystems) according to the manufacturer’s protocol. The amplification was performed following the conditions of 95°C for 10 min, 40 cycles at 95°C for 15 s, and 55°C–65°C for 1 min (with 0.3°C increase each cycle) and ending up with melting curve stage at 95°C for 15 s, 60°C for 1 min, and 95°C for 15 s to detect the presence of nonspecific products and primer dimers. The primers used are listed in **Supplementary Table S2**. The specificity of PCR products amplified were confirmed by the presence of a single peak in the dissociation melting curves and also visualized on agarose gel electrophoresis. Relative transcript expression levels were normalized in relation to the expression of the housekeeping gene Elongation factor 1 alpha (*elfα1*) (29), which did not vary between the samples. Expression levels of target genes in the treated group relative to the untreated/control group were calculated by subtracting the normalized Ct values of the treated group from that of the control groups (ΔΔCt). The 2^−ΔΔCt^ method (30) was used to determine the relative expression levels of the target genes.

### Laser Capture Microdissection

*B. belcheri* amphioxus were embedded in OCT compound (Sarura Finetek, Torrance, CA) and stored at −20°C. Frozen amphioxi were transversely sectioned (6 μm) using a cryostat microtome (Jung CM3000, Lieca Microsystems, Leitz, Germany) and mounted on plain slides. The Hatschek’s pit region and adjacent nerve cord (thickness 450–550 μm) were collected, and the tissue samples were stained with hematoxylin and eosin and visualized in the PixCell IIe Laser Capture Microdissection System (Arcturus, Sunnyvale, CA). Hatschek’s pit and nerve cord in the head of amphioxi were captured on CapSure HS laser capture microdissection (LCM) Caps (Arcturus).

### Paraffin Processing of Sexually Mature Amphioxus and Sectioning

Sexually mature amphioxus was cut into three pieces and fixed in 10% formalin in 100 mM phosphate-buffered saline (PBS, pH 7.4) at 4°C overnight. After, samples were dehydrated in graded ethanol (30%, 50%, 70%) and processed in Tissue Processor, Leica ASP300S. Samples were then treated with 95% ethanol for 1 h twice, followed by dehydration in 100% ethanol for 1 h twice and subsequently treated with toluene for 1 h twice before infiltrated with paraffin wax. The embedding of samples was performed with Embedding Centre, Leica EG1150. Paraffin blocks were sectioned at 8 μm, and the sections were mounted on HistoGrip™ (ThermoFisher) coated slides. After drying at 42°C overnight, the slides were stored at room temperature for further use.

### Immunofluorescence Staining

Paraffin sections to be stained were deparaffinized in xylene for 5 min three times and dehydrated in graded ethanol (100%, 95%, 70%, 50%, 30%). After washing for 5 min in 1× PBS, antigen of the samples was retrieved with boiling citrate buffer (pH 6.0) for 10 min and washed in 1× PBS for 5 min. For permeabilization, samples were incubated in 1× PBS with 0.1% Triton X-100 for 10 min and washed in 1× PBS for 5 min three times prior to the blocking step. For blocking, samples were incubated in 1× PBS with 5% BSA, 22.52 mg/ml glycine, and 0.1% Tween-20 for 1 h. Custom-made antibodies (Genscript, China) raised from rabbit were diluted in 1× PBS with 1% BSA, 22.52 mg/ml glycine, and 0.1% Tween-20 (final concentration 3 μg/ml) and samples incubated overnight at 4°C. On the following day, samples were washed in 1× PBS for 5 min three times prior to the addition of the secondary antibody (1:500), goat anti-Rabbit IgG (H+L) Highly Cross-Adsorbed Secondary Antibody-Alexa Fluor 594 (Invitrogen) and incubated for 1 h. Slides were washed with 1× PBS for 5 min three times and then stained with DAPI for 15 min. After washing, slides were mounted with Fluoro-Gel (Electron Microcopy Sciences) and the reactions were visualized on a Nikon 80i Fluorescent microscope, with Diagnostic Instrument Spot RT3 Slider.

### Probe Preparation and Cloning

Partial DNA fragments were amplified using specific primers (**Supplementary Table S3**) designed based on the predicted *B. belcheri* Bb_070560F sequence. The PCR reactions were performed as follows: 36 cycles at 94°C for 45 s, 50°C for 45 s, and 72°C for 90 s. The amplified products were ligated into pGEM-T Easy Vector (Promega). The recombinant vectors were linearized with the *ApaI* and *SacI* restriction enzymes to generate the sense or digoxigenin-labeled (DIG-labeled) antisense strand with SP6 or T7 RNA polymerase (Roche).

### *In Situ* Hybridization

Whole-mount *in situ* hybridization on embryos was conducted according to (31). For *in situ* hybridization on adult amphioxus, the tissues on slides were obtained as described above and incubated in xylene 5 min three times for deparaffinization following incubation in graded ethanol (100% ethanol × 2, 95%, 70%, 50%, and 30%, each for 3 min) for rehydrating the samples and washing step in 1× PBS. Samples were then permeabilized with 10 μg/ml Proteinase K in 100 mM Tris-HCl buffer (pH 8.0) with 50 mM EDTA at 37°C for 20 min and subsequently postfixed at room temperature for 20 min using 4% paraformaldehyde in 1× PBS (pH 7.4). The samples were incubated in pH 8.0 solution (0.9% NaCl, 0.1 M triethanolamine) for 5 min prior to 10 min acetylation by adding acetic anhydride (0.25%) into the pH 8.0 solution. Subsequently, samples were washed three times with 2× saline sodium citrate buffer (SSC) for 10 min. Next, prehybridization of samples were performed at 55°C for 1 h with hybridization buffer (50% (v/v) formamide, 0.6 M NaCl, 10 mM Tris‐HCl, 1× Denhardt’s solution, 1 mM EDTA, 100 μg/ml denatured UltraPure™ Herring Sperm DNA, and 50 μg/ml yeast tRNA, pH 7.5). After prehybridization, the sample was hybridized with 2 μl of *GHl* riboprobes (antisense or sense riboprobes) in prehybridization buffer at 50°C overnight in a humidified chamber. On the next day, samples were washed with 2× SSC at 50°C for 15 min three times and then treated with 20 μg/ml RNase A (in 2× SSC) for 30 min. The samples were then washed with 2× SSC for 15 min and three times with 0.5× SSC for 10 min. After washing, samples were incubated twice in buffer 1 (0.6 M NaCl, 0.4 M Tris‐base, pH 7.5) for 10 min prior to the blocking step in which samples were incubated in buffer 1 with 5% FBS and 0.1% Triton X‐100 for 90 min at room temperature. After blocking, samples were incubated overnight with anti‐DIG antibody (1:500) (Roche) in buffer 1 with 0.01% Triton X‐100 at 4°C. On the following day, samples were washed with buffer 1 for 10 min twice and then incubated in buffer 2 (0.1 M Tris‐base, 0.01 M NaCl, 0.05 M MgCl_2_, pH 9.5) for 10 min. Buffer 2 with 0.34 mg/ml NBT and 0.175 mg/ml 5‐bromo‐4‐chloro‐indolyl‐phosphate (BCIP) was used for color development, and Milli‐Q (MQ) H_2_O was used to stop color development. Samples were air dried prior to mounting.

### Peptides and Antibodies

The PACAP/GCGa, GCGb, and GCGc synthetic peptides (amidated at C-terminus) were purchased from Genscript Inc. (Piscataway, NJ), and purity was higher than 95.0%. Amphioxus-deduced mature peptide sequences were based on a previous report (9) and are listed in **Supplementary Table S4**. Polyclonal antibodies raised against BbPACAP/GCGa and BbGHl were outsourced to a company (GenScript) and were affinity purified. The peptide sequences used as antigens are listed in **Supplementary Table S5**. A cysteine was added to the N-terminus of the antigen and conjugated to keyhole limpet hemocyanin (KLH) for immunization. For negative controls, antibodies were preincubated overnight with the immunizing peptide in a 1:10 molar ratio, using standard preabsorption protocol.

### Experimental Salinity Challenge

*B. belcheri* (2.6–3.2 cm) taken from the maintaining tank (25 ppt) were divided in two groups. Nine amphioxi were transferred to a smaller container with 35 ppt ASW (prepared with 500 ml MQ) (experimental group), and the other 9 amphioxi were maintained at 25 ppt ASW (500 ml) (control group). Amphioxi were fed with the microalagae *Isochrysis galbana* (final concentration 1 × 10^4^ cells/ml) three times per day, and the ASW was changed every 24 h. Three amphioxi were then sampled from each container at 24, 48, and 72 h posttransferring. The heads were collected for total RNA extraction and RT‐qPCR for analyzing the expression of *PACAP/GCG-R*, *GHl*, *PACAP/GCGa*, and *PACAP/GCGb* and *PACAP/GCGc* transcripts as described above. The experiment was repeated twice, and no mortality was observed in the experimental and control tanks.

### PACAP/GCGa Peptide Treatment

Twelve amphioxi *B. belcheri* (2.6–3.2 cm) maintained in 35 ppt ASW for 72 h were transferred into six‐well plate (35 mm/well; Costar, San Diego, CA) and challenged with different concentrations of the PACAP/GCGa peptide. In each well, 3 ml of 35 ppt ASW with known concentration of PACAP/GCGa peptide was added, and animals were incubated for 30 min. The heads of amphioxi were collected to examine the changes in *GHl* expression. The experiment was repeated twice, and no mortality was observed. PACAP/GCGa peptide that led to significant upregulation of *GHl* transcript was further tested for a dose‐range (0.01, 0.03, 0.1, 0.3, and 1 μM) study. For each dose, three amphioxi were used, and the experiment was repeated twice.

## Results

### Isolation of Cephalochordate PACAP/GCG Peptide Precursors

**Figure 1:**
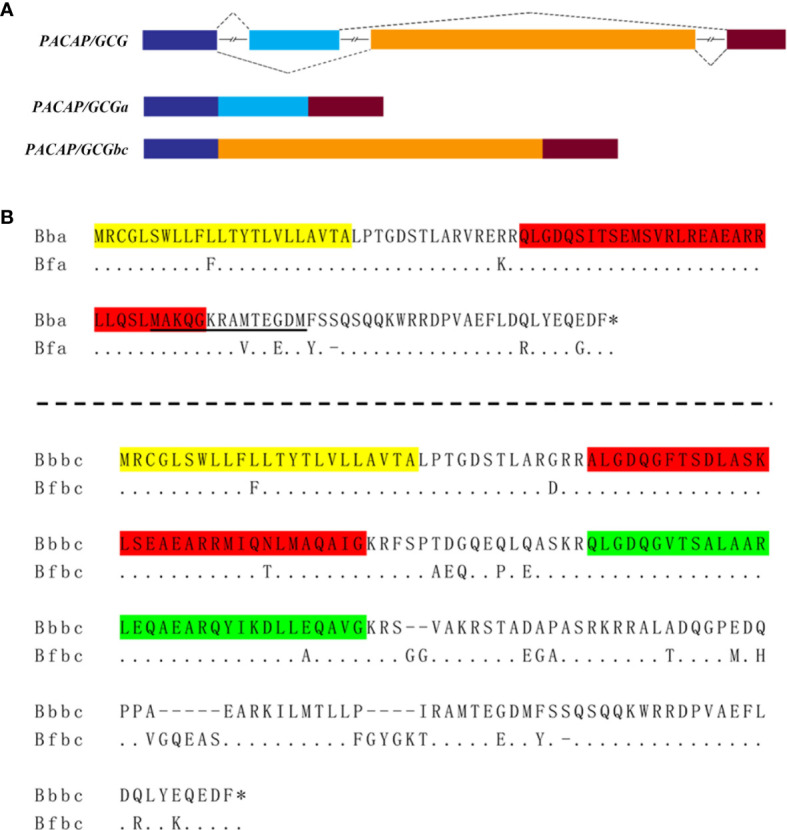
The amphioxi *PACAP/GCG* gene and transcripts. **(A)** Schematic representation of the *B*. *floridae* gene and the two alternative spliced transcript variants *PACAP/GCGa* and *PACAP/GCGbc*. The predicted exons are represented by different colored boxes, and the line indicates the introns. **(B)** Multiple sequence alignment of the deduced amino sequences of the two transcript precursor variants between *B. belcheri* (Bb) and *B. floridae* (Bf). The predicted signal peptide sequence is highlighted in yellow and the deduced mature peptides in each transcript precursor are in red (PACAP/CGCa and PACAP/GCGb) and green (PACAP/GCGc). The amino acid sequence underlined was used as antigen to produce the PACAP/GCGa antibody. The asterisk “^*^” represents the STOP codon. Bfa, *B. floridae PACAP/GCGa*; Bfbc, *B. floridae PACAP/GCGbc*; Bba, *B. belcheri PACAP/GCGa*; Bbbc, *B. belcheri PACAP/GCGbc*.

The putative *B. floridae* PACAP/GCG peptides were predicted by a previous study (9), and the corresponding gene sequence was retrieved from *B. floridae* genome database of (accession number 96553). The complete sequence of the *B. belcheri* homolog was isolated using a combination of RT-PCR and 5’ and 3’ RACE on cDNA and two full‐length transcripts were obtained, namely *PACAP/GCGa* and *PACAP/GCGbc* (**Supplementary Figures S1, S2**). For *in situ* hybridization, the coding domain sequence of their counterparts were also isolated from adult *B. floridae* cDNA (**Supplementary Figures S3, S4**) and a multiple sequence alignment of their nucleotide sequences with the corresponding gene showed that they correspond to two alternative splicing variants of the same locus. The amphioxi *PACAP/GCG* gene is composed of four exons and is spliced into *PACAP/GCGa* containing the first, second, and the fourth exons and *PACAP/GCGbc* transcript that shares the first and last exons with *PACAP/GCGa* transcript but possess the gene third exon (**Figure 1**). Alignment of the *B. belcheri-* and *B. floridae*-predicted amino sequences showed a high sequence conservation especially within the region encoding the mature peptide sequences and the *B. floridae* and *B. belcheri* PACAP/GCGa-deduced mature peptides are 100% identical and the other share >96% amino acid sequence similarity.

### Mapping of PACAP/GCG System in Amphioxus

**Figure 2:**
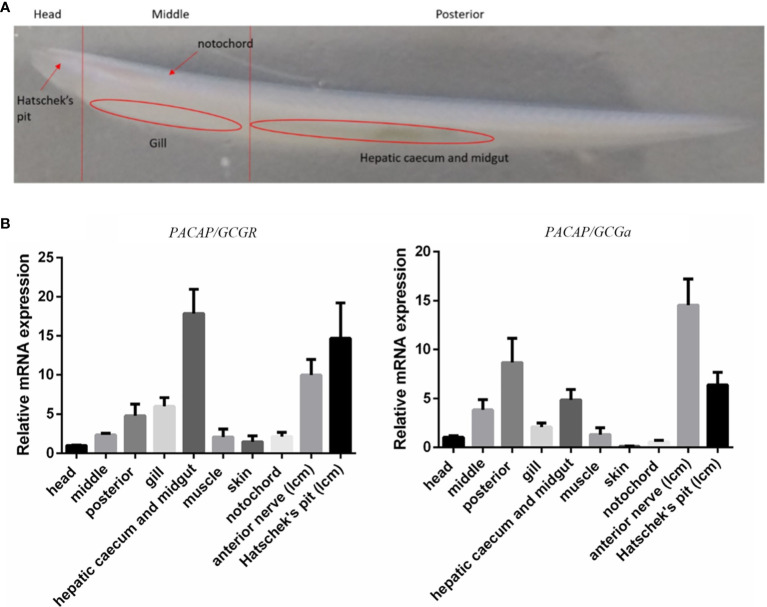
Expression profile of the *PACAP/GCGa* and *PACAP/GCG-R* transcripts in adult *B*. *belcheri*. **(A)** Representative image of a whole adult individual indicating the localization of the collected tissues for expression analysis. **(B)** Relative expression of *PACAP/GCGa* and *PACAP/GCG-R* in the head (without the gills), middle part (without hepatic caecum), posterior part (with hepatic caecum and midgut), gills, hepatic caecum and midgut, muscle, skin, notochord, anterior nerve (LCM), and Hatschek’s pit (LCM). Melting curves for each transcript in qRT-PCR are all a single peak. Data is expressed as means ± SEM (*n* = 6).

To characterize the tissue distribution of the *PACAP/GCG* transcripts and its cognate receptor, different strategies were used from embryo to adult. During *B. floridae* embryogenesis, whole-mount *in situ* hybridization (WISH) was conducted to detect the *PACAP/GCGa* and *PACAP/GCGbc* precursors and showed that their expression all initiate during T0 and L0 stages (**Supplementary Figures S5, S6**), but they show a differential expression pattern. For the *PACAP/GCGa*, three positive cells were detected in the posterior cerebral vesicle, of which the two with round shape were distributed one on the *B. floridae* larva left body side and the other on the right body side (**Supplementary Figure S6**). In subsequent L0 stage, three cells were also stained in the posterior cerebral region, with several extra *PACAP/GCGa*-positive cells located anterior to the first pigment spot, of which two were observed on the larva right body side and one on the left body side (**Supplementary Figure S7**). During the L1 stage, the distribution of *PACAP/GCGa*-positive cells resembled that of L0 stage, with an extra positive cell observed in the region attributed as the primary motor center (PMC) (**Supplementary Figure S8**). For the *PACAP/GCGbc* probe, positive cells were first observed in the anterior end of the cerebral vesicle very close to the neuropore and the future rostral pigment spot (**Supplementary Figure S9**). At the L0 stage, positive cells persist in the same region where the two positive cells were identified on the right side near the neuropore and a cell in the posterior cerebral vesicle (**Supplementary Figure S9**). At L1 stage, the positive signal near the anterior end of the cerebral vesicle was not identified but the signal in the posterior cerebral vesicle is still observed (**Supplementary Figure S9**). At the same developmental stage, two positive cells with strong signal were also identified in the region anterior to the first pigment spot, one on each side of the larva (**Supplementary Figure S9**). In adult *B. belcheri* amphioxus, the tissue distribution of the two peptide precursors and cognate receptor were analyzed by qRT-PCR (**Figure 2**). In general, expression of *PACAP/GCGa* and receptor were detected in most of the tissues analyzed and *PACAP/GCGa* was highest in the anterior nerved followed by Hatscheck’s pit and in the posterior amphioxus region which includes the gut, followed by expression in the middle region which is mostly covered by the gill and related structures (**Figure 2B**). The *PACAP/GCG-R* was also most abundant in the nerve cord and Hatschek’s pit and also in the hepatic caecum and midgut (**Figure 2B**). The high expression level of both transcripts in the anterior nerve as well as in the Hatschek’s pit and their relatively low abundance in the head region imply for their restricted tissue expression pattern. Furthermore, expansion of the *PACAP/GCGa* expression area from limitation at neural tube in the embryo to Hatschek’s pit, gill, and gut in the adult amphioxi is suggestive of potential pleiotropic roles.

### Detection of PACAP/GCGa by Immunofluorescence

**Figure 3:**
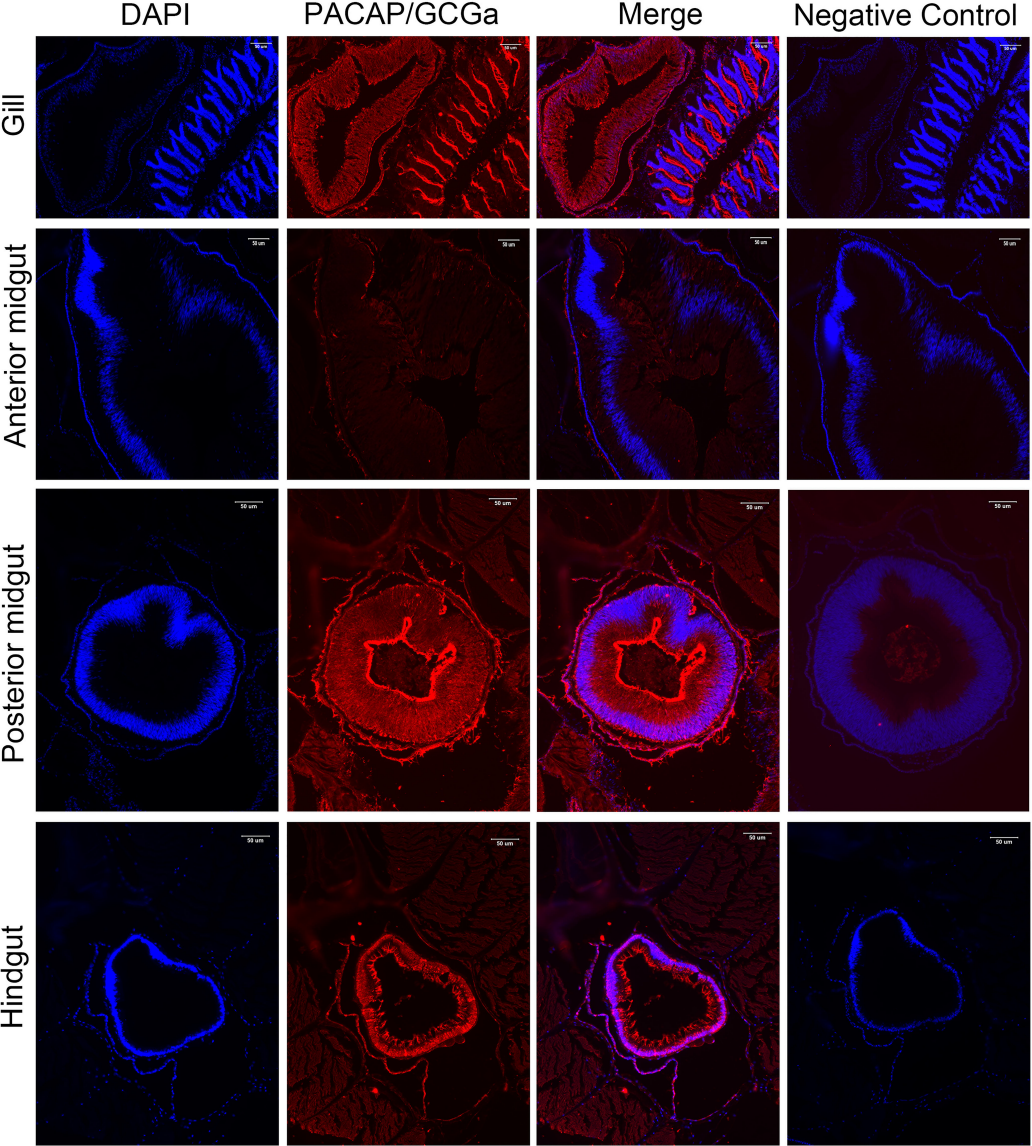
Detection of PACAP/GCGa in several adult *B*. *belcheri* tissues by immunofluorescence. Immunofluorescence digital images of DAPI staining (blue), PACAP/GCGa (red) positive signals, and merged and negative control (merged). Negative controls represent the result of incubations in which primary antibody with overnight incubation with antigen in 1:10 molar ratio was used to replace primary antibody alone. PACAP/GCGa peptides were detected in the gill, midgut, and hindgut. N, notochord; Hp, Hatschek’s pit. The scale bar is 50 μm and is indicated in the figures.

**Figure 4:**
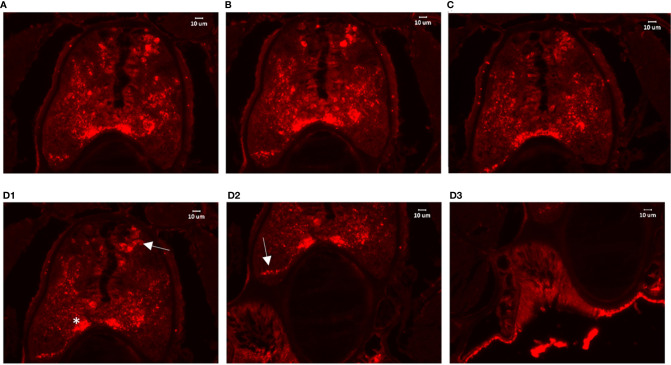
Detection of PACAP/GCGa in the anterior nerve cord of *B*. *belcheri* after high salinity challenge. Animals were exposed to 35 ppt ASW for 72 h The digital images represent a series of 4 consecutive transverse sections (from **(A)** he anterior part to **(D)** posterior part) within the Hatschek’s pit region. **(D)** Representative images for PACAP/GCGa distribution on different regions of the same section **(D1–D3)**. In **(D1)**, the arrow and asterisk indicate intense staining reaction in the dorsal Joseph cells and in the region close to the nucleus of Rodhe. The arrow in **(D2)** indicates the intense staining reaction in the infundibulum-like downgrowth approaching the Hatschek’s pit. The scale bar is 10 μm and is indicated in the figures.

**Figure 5:**
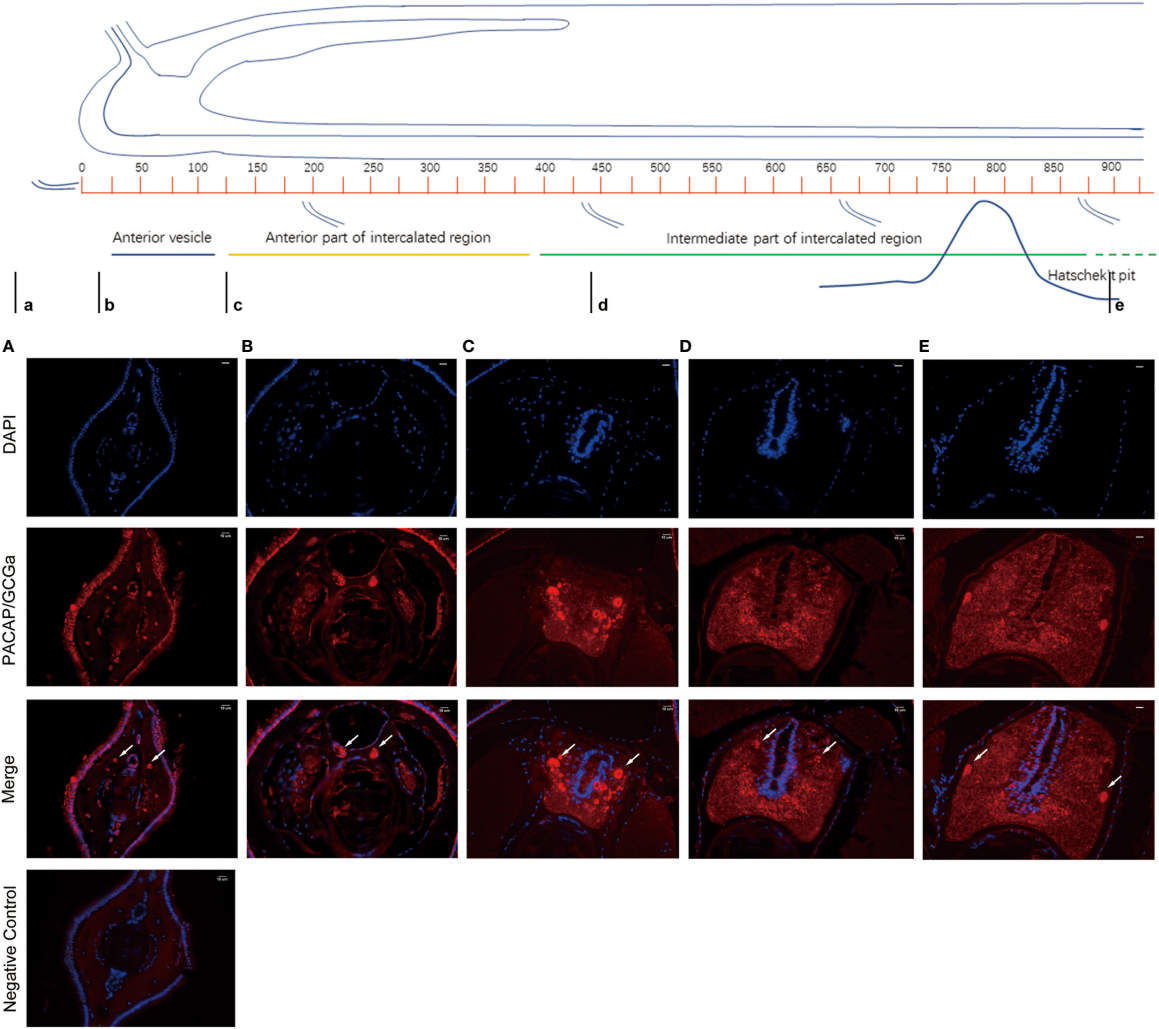
Detection of PACAP/GCGa in *B*. *belcheri* by immunofluorescence staining in the anterior nerve cord. The anterior brain of an adult amphioxus is represented on the top [adapted from Castro et al. 2015 (33)]. The small letters below the image indicate the location of the transversion sections analyzed **(A–E)**. Immunofluorescence staining digital images of DAPI (blue) and PACAP/GCGa (red) and the merged anterior nerve cord region sections. The white arrows indicate the condensed points of antibody positive signal against PACAP/GCGa in *B*. *belcheri* neurons. The scale bar is 10 μm and is indicated in the figures.

To colocalize PACAP/GCGa precursor and its endogenous protein, a custom polyclonal antibody against PACAP/GCGa peptide was used for immunofluorescence staining. Our data indicated the presence of PACAP/GCGa precursor and/or peptide in the region of *B. belcheri* nerve cord, gill bar, Hatschek’s pit, and epithelial lining of the digestive tract (both midgut and hindgut) highlighting for its potential importance in the amphioxi nervous system (**Figure 3** and **Supplementary Figure S10**). Interestingly, the immunoreactivity against PACAP/GCGa precursor and/or peptide gradually increased from the anterior midgut (hardly detected) to posterior midgut, but this was not detected by qRT-PCR (**Figure 2B**). Although we have not use qRT-PCR to measure *PACAP/GCGa* expression in the hindgut, a positive signal was detected. High positive reactions were also detected in the posterior intercalated region of the amphioxus anterior nerve cord and around the Rohde nucleus cells located ventral to the central canal along the nerve cord that coincide with the presence of Hatschek’s pit and its related structure, wheel organ, in the roof of the oral cavity (**Figure 4** and **Supplementary Figure S10**). Our finding supports the hypothesis that the nucleus of Rohde cells are homologous to the neurosecretory cells in the vertebrate hypothalamus region (32) where PACAP is highly abundant (22, 23). Stained PACAP/GCGa-positive cells were also detected downgrowth of the nerve cord approaching the Hatschek’s pit which might come from the nucleus of the Rohde cells (**Figure 4**). Moreover, the detected signal in neuron axons of the nerve cord suggested that *PACAP/GCGa* in amphioxus is likely to function as neurotransmitter (**Figure 5**). In the lamprey (*Petromyzon marinus*), communication between adenohypophysis and neurohypophysis can be made *via* diffusion (34), and in amphioxus, Gorbman in 1999 (35) also suggested that a similar communication between CNS and Hatschek’s pit *via* infundibulum-like downgrowth by diffusion can occur. This was also supported by our results as presence of PACAP/GCGa-precursor immunoreactivities in downgrowth infundibulum of the nerve cord was observed (**Figure 4**). It is possible that the nucleus of Rohde in amphioxus diffuse neurosecretory peptides into Hatschek’s pit to regulate its activities. The detection of PACAP/GCGa peptide or precursor in dorsal Joseph cells, the melanopsin-expressing photoreceptors (36) was apparent (**Figure 4**), and this is consistent with the exclusive distribution of melanopsin in the PACAP-containing retinal ganglion cells of the rat retinohypothalamic tract (37).

### GH-Like Hormone Regulation by PACAP/GCGa Peptide Under a High Salinity Challenge

**Figure 6:**
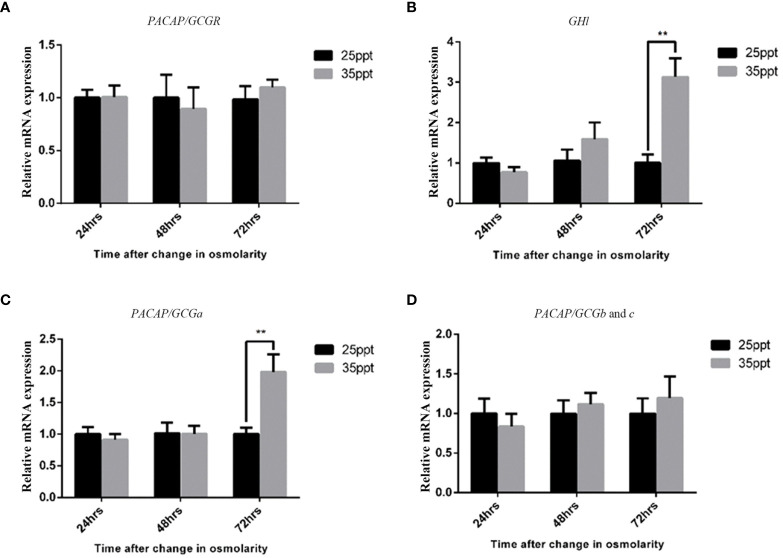
Effect of high salinity challenge in the expression of the *PACAP/GCG* system and *GHl* in the head of *B*. *belcheri*. Gene expression levels of **(A)** *PACAP/GCG-R*, **(B)** GHl, **(C)** *PACAP/GCGa*, and **(D)** *PACAP/GCGb* and *PACAP/GCGc* determined by quantitative PCR and normalized using the *ef1a* expression as reference. Data represent the pool of samples collected from two independent experiments (*n* = 3/experiment) and is expressed as means ± SEM (*n* = 6 unpaired *t*-test, ^**^p ≤ 0.01).

**Figure 7:**
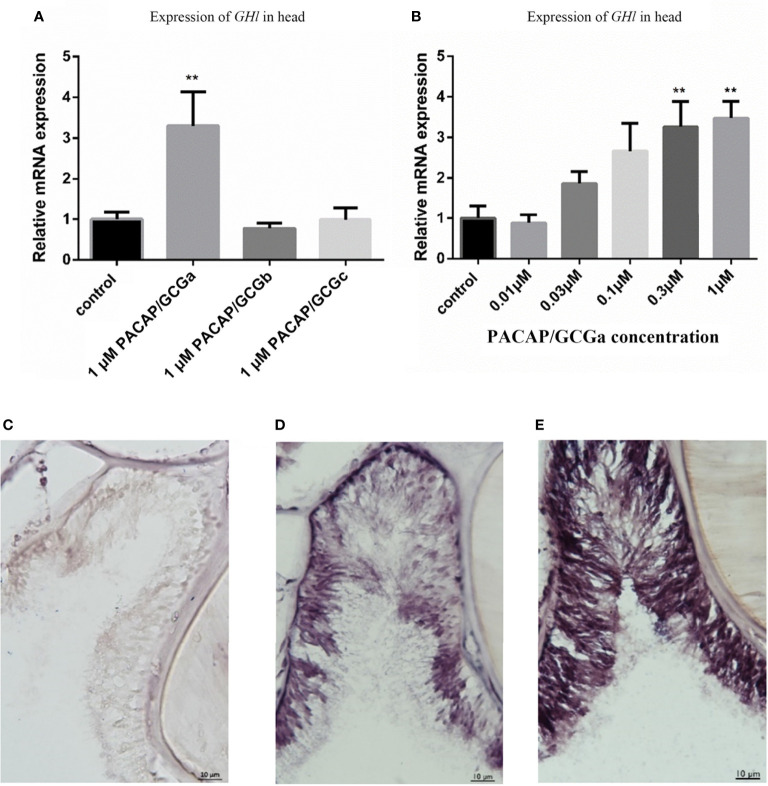
Stimulation of *GHl* expression by exposing *B*. *belcheri* to PACAP/GCGa peptide. **(A)** Potency of the three cephalochordate PACAP/GCGs peptides in *GHl* expression. The synthetic *B. floridae*-predicted peptides were used, and they share >96% amino acid sequence similarity with *B*. *belcheri* orthologs. *B*. *belcheri* PACAP/GCGa (BbPACAP/GCGa) is 100% identical in amino acid sequence to *B*. *floridae* ortholog (BfPACAP/GCGa). Gene expression levels were determined by quantitative PCR and normalized using the *ef1a* expression as reference. **(B)** Changes of *GHl* gene expression levels to different concentrations (0.01 to 1 μM) of BbPACAP/GCGa peptide. Data represent the result of two independent experiments (*n* = 3/experiment) and are expressed as means ± SEM. Statistical significant differences were assessed using one‐way ANOVA followed by the Dunnett’s test. ^**^p ≤ 0.001. **(C–E)** Digital images showing *GHl* expression detected by *in situ* hybridization in the *B. belcheri* Hatschek’s pit: **(C)** negative control, **(D)** after high salinity challenge (35 ppt for 72 h), and **(E)** after high salinity challenge and exposure to 1 μM BfPACAP/GCGa. Intense staining was observed in the presence of the BfPACAP/GCGa peptide. The scale bar is 10 μm and is indicated in the images.

**Figure 8:**
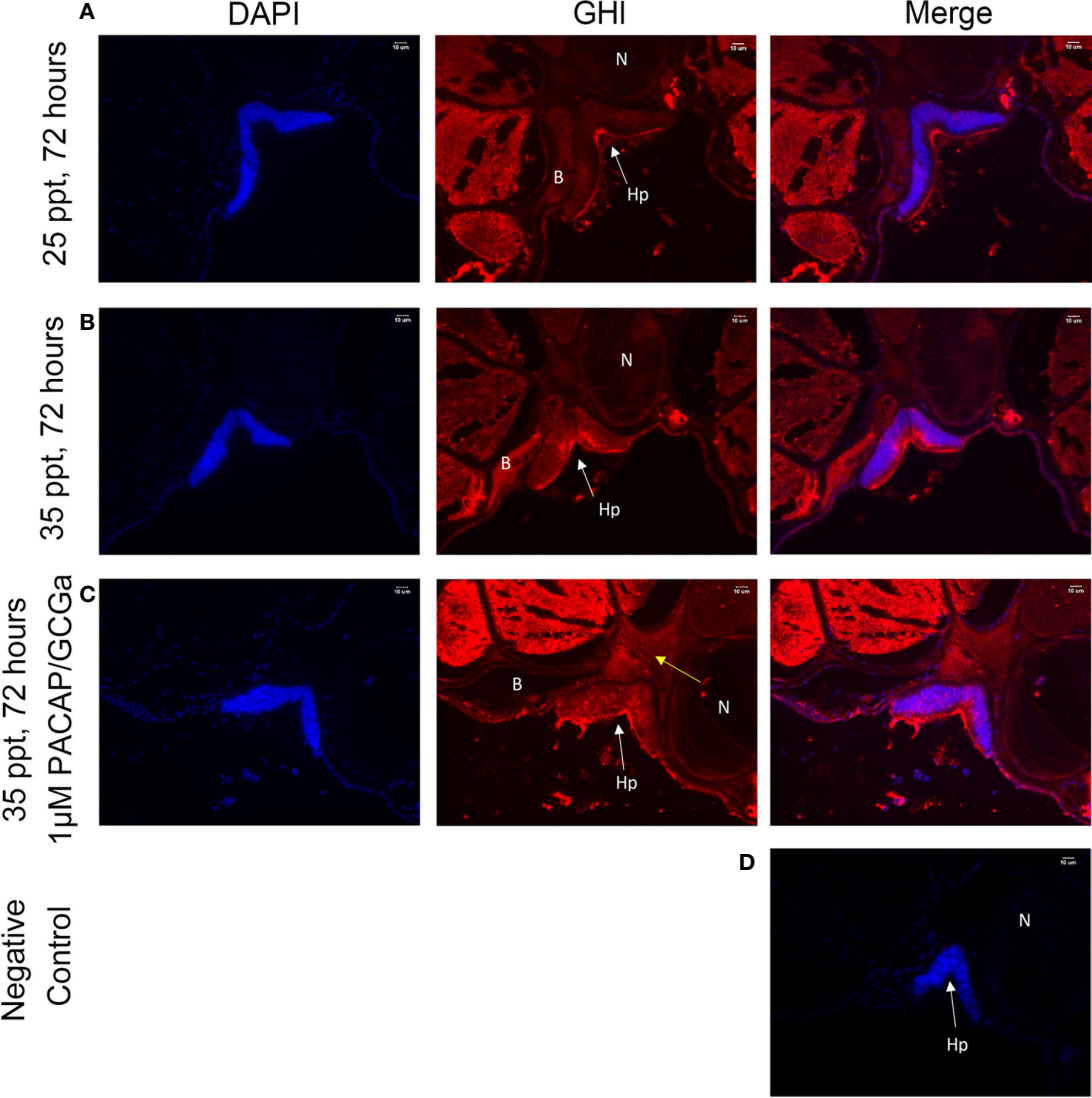
GHl-positive signals in the *B*. *belcheri* Hatschek’s pit by immunofluorescence staining. Immunofluorescence digital images of DAPI staining (blue) and GHl-positive (red) signals and the merged and negative control (merged). The location of GHl in Hatschek’s pit was detected using a polyclonal antibody against *B*. *belcheri* GHl. Digital images of the **(A)** control at 25 ppt ASW (control), **(B)** after 72 h exposure to 35 ppt ASW, and **(C)** after exposure to 1 μM BfPACAP/GCGa. White arrows indicate the positively stained GHl (red) near the blood cavity, and the yellow arrows indicate the positive signals in the connective tissue (acellular) and in the blood cavity. DAPI staining is in blue. **(D)** Negative control. N, notochord; Hp, Hatschek’s pit; B, blood cavity region. The scale bar is 10 μm and is indicated in the images.

Previously, the GH-like hormone, a functional homolog of the vertebrate growth hormone as well as its binding protein, were identified in amphioxus (11), and it was demonstrated that GH-like hormone increases in Hatschek’s pit reacting to high salinity in a manner resembling the mechanisms of teleost GH (26). Given that the regulation of GH in vertebrates is generally controlled by PACAP subfamily peptides (38–42), we then examined the relationship between PACAP/GCGs and GHl in amphioxus. After high salinity treatment (35 ppt), a 3-fold increase of the *GHl* expression in the cephalic zone of amphioxus was observed when compared with the control (**Figure 6**), and this was confirmed by the immunofluorescence detection of GHl peptide in the Hatschek’s pit (**Figure 8**). *GHl* expression was mainly observed in the lateral region of the pit (type 1 Hatschek’s pit groove cells), where loosened collagen layers were observed (43) (**Figures 7C, D**). Upregulation of *PACAP/GCGa* transcript level in the head region was also observed, but no changes in the expression of *PACAP/GCGb* and *PACAP/GCGc* and *PACAP/GCG-R* were detected (**Figure 6**). Immunofluorescence staining showed an increase of PACAP/GCGa detection in the Hatschek’s pit after high salinity challenge (**Supplementary Figure S10**). This increase in expression was observed in wheel organ cells, Hatschek’s pit groove types 1 and 2 cells, whereas PACAP/GCGa level in the margin cells is generally high in both 25- and 35-ppt samples. The staining intensity also increased in dorsal Joseph cells, the region closer to the nucleus of Rohde and the downgrowth infundibulum of the nerve cord (**Supplementary Figure S10**). To investigate if *PACAP/GCGa* regulates *GHl* expression, we treated the animals with the PACAP/GCGa peptide 72 h after the high salinity challenge. Taking advantage of Hatschek’s pit’s location in the oral cavity, treatment was performed by immersing amphioxus in artificial seawater with the peptide. Only the BfPACAP/GCGa peptide was able to upregulate *GHl* expression in the cephalic area (**Figure 7A**), and this was dose dependent and statistically significant at the higher doses tested (0.3 and 1 μM, *p* ≤ 0.001) in comparison with the control (**Figure 7B**). This was confirmed by WISH and treatment of 1 μM PACAP/GCGa peptide led to an augmentation of *GHl* expression in the Hatschek’s pit (**Figures 7C–E**). Consistent with the WISH results, immunofluorescence staining showed that after PACAP/GCGa treatment, *GHl* expression level in the Hatschek’s pit increased and appeared to be released into the connective tissues immediately adjacent to the blood cavity (**Figure 8**). This suggests that amphioxus can respond to high salinity challenge through releasing GHl from Hatschek’s pit into tissues under the regulation of *PACAP/GCGa* peptide.

## Discussion

In this study, we isolated the cephalochordate *PACAP/GCG* transcripts and provide expression data of their function in the CNS-Hatschek’s pit axis. Previously, PACAP cDNAs that share high sequence homology with that of the vertebrates have been isolated from other invertebrates including deuterostomes such as tunicates, but their existence in the genomes and transcriptomes remains to be confirmed (13). The cephalochordate PACAP/GCG gene encodes for three mature peptides that probably evolved by exon duplication (44) and share limited amino acid sequence similarity with the vertebrate ortholog (9, 13, 18, 19, 44, 45). Nonetheless, they were able to activate the amphioxi sequence ortholog of the vertebrate PACAP receptor but not human VPAC_2_R, PAC_1_R, and GCGR, suggesting that structural differences between the amphioxi PACAP/GCGs and vertebrate PACAP and VIP peptides may exist (12, 44). The amphioxi gene produced two transcript isoforms with different coding potentials. We have isolated a *PACAP/GCGa* precursor that encodes PACAP/GCGa and the *PACAP/GCGbc* precursor that encodes both PACAP/GCGb and PACAP/GCGc peptides, and they seemed to have different functions (9, 13). In the amphioxus *B. floridae*, both transcripts have a similar expression during early embryonic development and are limited to the neural tube, but *PACAP/GCGa* precursor was mostly expressed in the posterior cerebral vesicle. In adult amphioxus, expression of *PACAP/GCGa* expands and was detected in the Hatschek’s pit, gill, and gut, suggestive of a potential pleiotropic roles as described for the orthologs in vertebrates (46, 47). The intense staining signal in neuronal axons in the nerve cord also suggests that similar to the vertebrate PACAP, the amphioxus *PACAP/GCGa* likely functions as a putative neurotransmitter (9, 12). The reaction of the cephalochordate *PACAP/GCGa* to high salinity challenge *via* changing the regulation of *GHl* expression resembles that of the vertebrate PACAP and sequence-related peptides of the Secretin subfamily (38–42), revealing that the cephalochordate prototypical system function was maintained during the vertebrate evolution.

In the adult amphioxus, the Hatschek’s pit, a structure which resides asymmetrically in the roof of oral cavity, is considered to be the functional homolog of the vertebrate pituitary. Tjoa et al. (48) using electron microscopy identify peptidergic granules in cephalochordate Hatschek’s pit and suggested for the first time the potential secretory function of this structure. The Hatschek’s pit is connected to the amphioxus CNS by an infundibulum-like extension from the anterior nerve cord (35). Its development is similar to the Rathke’s pouch that gives rise to the anterior pituitary in the vertebrate embryo (49). Moreover, the presence of prolactin (50) and gonadotropin-immunoreactivity (51) and the restricted expression of transcription factors *pit-1* in the embryonic primordium of Hatschek’s pit in cephalochordate have been reported (52). Nonetheless, the connection of Hatschek’s pit with the endocrine system and its potential neuroendocrine role which are key functions of the vertebrate pituitary remained to be demonstrated.

Up to now, homologs of the vertebrate HP axis peptides including kisspeptin (8), gonadotropin-releasing hormone-like (5), thyrostimulin (3), vasotocin (3), PACAP/GCGs (12), and growth hormone-like protein (GHl) (11) have been described in the amphioxus. Expression of thyrostimulin and vasotocin was not detected in the Hatschek’s pit by *in situ* hybridization, and Kubokawa et al. (3) suggested that reproduction of amphioxus could be controlled directly by neuroendocrine substance(s) from the nerve cord instead of pituitary-like structure. Likewise, immunoreactivity of the gonadotropin-releasing hormone-like protein was also restricted to the region near the central canal of the nerve cord in the amphioxus. However, a subsequent study demonstrated that the kisspeptin-like peptide in the amphioxus, which stimulates thyrostimulin expression detected in Hatschek’s pit as well as in the nerve cord leading to the hypothesis that Hatschek’s pit may function as a pituitary-like unit in the regulation of amphioxus reproductive axis (8).

In amphioxus, the GHl is considered the only classic pituitary hormone predominantly expressed in Hatschek’s pit (11), and this was confirmed using immunofluorescence staining. *GHl* expression was stimulated by increased water salinity (26). In vertebrates, PACAP subfamily members, including GHRH, PACAP, and PRP regulate GH releasing from the pituitary gland (39–42, 53–55), and we also demonstrated that increased expression of *GHl* in the amphioxus head is accompanied by increased PACAP/GCGa peptide concentration. This suggests that the regulation of PACAP/GCGa on GHl mimics what has been shown with PACAP and GH in the vertebrates (39–42, 53–55). Immunofluorescence staining also detected diffusion of GHl from Hatschek’s pit into the connective tissues close to the blood cavity in the PACAP/GCGa-treated animals, suggesting that Hatschek’s pit may have endocrine functions (10, 35) (**Figure 8C**). In addition, a PACAP/GCGa strong signal was observed in the area ventral to central canal of the nerve cord in the posterior intercalated region where connection between Hatschek’s pit and nerve cord occurs, which may support the hypothesis that the infundibulum may resemble the vertebrate neurohypophysis (10, 35). In conclusion, the CNS-Hatschek’s pit structure and the Hatschek’s pit are likely to be the homolog of HP axis and pituitary of vertebrates, respectively, and the CNS-Hatschek’s pit axis may lay the foundation of HP system in vertebrate, providing strong insights into origin and evolution.

## Data Availability Statement

The original contributions presented in the study are included in the article/**Supplementary Material**. Further inquiries can be directed to the corresponding author.

## Ethics Statement

Ethics review and approval was not required as per local legislation and institutional requirements.

## Author Contributions

The corresponding authors are responsible for the authenticity of the data. All authors made a contribution to the work reported, i.e., JO contributed to the conception design, execution of the study, acquisition, analysis, or interpretation of the data. LS contributed to the data organization, manuscript drafting, and revision. HS and AA engaged in partial experiments. GL and JC contributed to the revision and critical review of the article. All authors approved the final version of the manuscript to be published and agreed regarding the journal to which it has been submitted. All authors have agreed to be accountable for all aspects of the work.

## Funding

The work is supported by Hong Kong Government GRF 17112317, CRCG 201811159257, NSFC/GRF N_HKU742/20, to BKCC, NSFC-RGC 32061160471, the Natural Science Foundation of Fujian Province of China 2019J01022, the Youth Innovation Fund Project of Xiamen 3502Z20206032 to GL, and the Portuguese Foundation for Science and Technology (FCT) Project UIDB/04326/2020 to JCRC.

## Conflict of Interest

The authors declare that the research was conducted in the absence of any commercial or financial relationships that could be construed as a potential conflict of interest.

## Publisher’s Note

All claims expressed in this article are solely those of the authors and do not necessarily represent those of their affiliated organizations, or those of the publisher, the editors and the reviewers. Any product that may be evaluated in this article, or claim that may be made by its manufacturer, is not guaranteed or endorsed by the publisher.

## References

[1] Sower SA. Landmark Discoveries in Elucidating the Origins of the Hypothalamic-Pituitary System From the Perspective of a Basal Vertebrate, Sea Lamprey. Gen Comp Endocrinol (2018) 264:3–15. doi: 10.1016/j.ygcen.2017.10.016 29111305

[2] Kawauchi H Sower SA. The Dawn and Evolution of Hormones in the Adenohypophysis. Gen Comp Endocrinol (2006) 148(1):3–14. doi: 10.1016/j.ygcen.2005.10.011 16356498

[3] Kubokawa K Tando Y Roy S. Evolution of the Reproductive Endocrine System in Chordates. Integr Comp Biol (2010) 50(1):53–62. doi: 10.1093/icb/icq047 21558187

[4] Hwang JI Moon MJ Park S Kim DK Cho EB Ha N. Expansion of Secretin-Like G Protein-Coupled Receptors and Their Peptide Ligands *via* Local Duplications Before and After Two Rounds of Whole-Genome Duplication. Mol Biol Evol (2013) 30(5):1119–30. doi: 10.1093/molbev/mst031 23427277

[5] Roch GJ Tello JA Sherwood NM. At the Transition From Invertebrates to Vertebrates, a Novel GnRH-Like Peptide Emerges in Amphioxus. Mol Biol Evol (2014) 31(4):765–78. doi: 10.1093/molbev/mst269 PMC3969558 24361996

[6] On JSW Chow BKC Lee LTO. Evolution of Parathyroid Hormone Receptor Family and Their Ligands in Vertebrate. Front Endocrinol (2015) 6:28. doi: 10.3389/fendo.2015.00028 PMC4354418 25806022

[7] Holland LZ Albalat R Azumi K Benito-Gutierrez E Blow MJ Bronner-Fraser M. The Amphioxus Genome Illuminates Vertebrate Origins and Cephalochordate Biology. Genome Res (2008) 18(7):1100–11. doi: 10.1101/gr.073676.107 PMC2493399 18562680

[8] Wang P Wang M Ji GD Yang SS Zhang SC Liu ZH. Demonstration of a Functional Kisspeptin/Kisspeptin Receptor System in Amphioxus With Implications for Origin of Neuroendocrine Regulation. Endocrinology (2017) 158(5):1461–73. doi: 10.1210/en.2016-1848 28324048

[9] Mirabeau O Joly JS. Molecular Evolution of Peptidergic Signaling Systems in Bilaterians. Proc Natl Acad Sci USA (2013) 110(22):E2028–37. doi: 10.1073/pnas.1219956110 PMC3670399 23671109

[10] Gorbman A. Brain-Hatschek's Pit Relationships in Amphioxus Species. Acta Zoologica (1999) 80(4):301–5. doi: 10.1046/j.1463-6395.1999.00027.x

[11] Li MY Gao Z Ji DR Zhang SC. Functional Characterization of GH-Like Homolog in Amphioxus Reveals an Ancient Origin of GH/GH Receptor System. Endocrinology (2014) 155(12):4818–30. doi: 10.1210/en.2014-1377 25333966

[12] On JSW Duan C Chow BKC Lee LTO. Functional Pairing of Class B1 Ligand-GPCR in Cephalochordate Provides Evidence of the Origin of PTH and PACAP/Glucagon Receptor Family. Mol Biol Evol (2015) 32(8):2048–59. doi: 10.1093/molbev/msv087 PMC4833073 25841489

[13] Cardoso JCR Garcia MG Power DM. Tracing the Origins of the Pituitary Adenylate-Cyclase Activating Polypeptide (PACAP). Front Neurosci (2020) 14:366. doi: 10.3389/fnins.2020.00366 32508559 PMC7251081

[14] Sun CH Song DY Davis-Taber RA Barrett LW Scott VE Richardson PL. Solution Structure and Mutational Analysis of Pituitary Adenylate Cyclase-Activating Polypeptide Binding to the Extracellular Domain of PAC1-Rs. Proc Natl Acad Sci USA (2007) 104(19):7875–80. doi: 10.1073/pnas.0611397104 PMC1876540 17470806

[15] Bourgault S Vaudry D Segalas-Milazzo I Guilhaudis L Couvineau A Laburthe M. Molecular and Conformational Determinants of Pituitary Adenylate Cyclase-Activating Polypeptide (PACAP) for Activation of the PAC1 Receptor. J Medicinal Chem (2009) 52(10):3308–16. doi: 10.1021/jm900291j 19413310

[16] Dejda A Bourgault S Doan ND Letourneau M Couvineau A Vaudry H. Identification by Photoaffinity Labeling of the Extracellular N-Terminal Domain of PAC1 Receptor as the Major Binding Site for PACAP. Biochimie (2011) 93(4):669–77. doi: 10.1016/j.biochi.2010.12.010 21185349

[17] Cardoso JC Vieira FA Gomes AS Power DM. PACAP, VIP and Their Receptors in the Metazoa: Insights About the Origin and Evolution of the Ligand–Receptor Pair. Peptides (2007) 28(9):1902–19. doi: 10.1016/j.peptides.2007.05.016 17826180

[18] Cardoso JC Vieira FA Gomes AS Power DM. The Serendipitous Origin of Chordate Secretin Peptide Family Members. BMC Evol Biol (2010) 10:135. doi: 10.1186/1471-2148-10-135 20459630 PMC2880984

[19] Sherwood NM Krueckl SL McRory JE. The Origin and Function of the Pituitary Adenylate Cyclase-Activating Polypeptide (PACAP)/glucagon Superfamily. Endocrine Rev (2000) 21(6):619–70. doi: 10.1210/edrv.21.6.0414 11133067

[20] Vaudry D Falluel-Morel A Bourgault S Basille M Burel D Wurtz O. Pituitary Adenylate Cyclase-Activating Polypeptide and its Receptors: 20 Years After the Discovery. Pharmacol Rev (2009) 61(3):283–357. doi: 10.1124/pr.109.001370 19805477

[21] Vaudry D Gonzalez BJ Basille M Yon L Fournier A Vaudry H. Pituitary Adenylate Cyclase-Activating Polypeptide and its Receptors: From Structure to Functions. Pharmacol Rev (2000) 52(2):269–324. doi: 10.1124/pr.109.001370 10835102

[22] Arimura A Somogyvarivigh A Miyata A Mizuno K Coy DH Kitada C. Tissue Distribution of Pacap as Determined by Ria - Highly Abundant in the Rat-Brain and Testes. Endocrinology (1991) 129(5):2787–9. doi: 10.1210/endo-129-5-2787 1935809

[23] Ghatei MA Takahashi K Suzuki Y Gardiner J Jones PM Bloom SR. Distribution, Molecular Characterization of Pituitary Adenylate Cyclase-Activating Polypeptide and Its Precursor Encoding Messenger RNA in Human and Rat Tissues. J Endocrinol (1993) 136(1):159–66. doi: 10.1677/joe.0.1360159 8094091

[24] Hirabayashi T Nakamachi T Shioda S. Discovery of PACAP and its Receptors in the Brain. J Headache Pain (2018) 19(1):1–8. doi: 10.1186/s10194-018-0855-1 29619773 PMC5884755

[25] Warfvinge K Edvinsson L. Cellular Distribution of PACAP-38 and PACAP Receptors in the Rat Brain: Relation to Migraine Activated Regions. Cephalalgia (2020) 40(6):527–42. doi: 10.1177/0333102419893962 31810401

[26] Li M Jiang C Zhang Y Zhang S. Activities of Amphioxus GH-Like Protein in Osmoregulation: Insight Into Origin of Vertebrate GH Family. Int J Endocrinol (2017) 2017:9538685. doi: 10.1155/2017/9538685 28408927 PMC5376476

[27] Li G Shu Z Wang Y. Year-Round Reproduction and Induced Spawning of Chinese Amphioxus, Branchiostoma Belcheri, in Laboratory. PloS One (2014) 9(5):e99264. doi: 10.1371/journal.pone.0099264 PMC3784433 24086537

[28] Li G Yang X Shu ZH Chen XY Wang YQ. Consecutive Spawnings of Chinese Amphioxus, Branchiostoma Belcheri, in Captivity. PloS One (2012) 7(12):e50838. doi: 10.1371/journal.pone.0050838 23251392 PMC3520940

[29] Wang Y Zhang S. Ef1α Is a Useful Internal Reference for Studies of Gene Expression Regulation in Amphioxus Branchiostoma Japonicum. Fish Shellfish Immunol (2012) 32(6):1068–73. doi: 10.1016/j.fsi.2012.03.001 22554576

[30] Livak KJ Schmittgen TD. Analysis of Relative Gene Expression Data Using Real-Time Quantitative PCR and the 2(T)(-Delta Delta C) Method. Methods (2001) 25(4):402–8. doi: 10.1006/meth.2001.1262 11846609

[31] Yu JK Holland LZ. Amphioxus Whole-Mount in Situ Hybridization. Cold Spring Harb Protoc (2009) 2009(9):pdb prot5286. doi: 10.1101/pdb.prot5286 20147271

[32] Wicht H Lacalli TC. The Nervous System of Amphioxus: Structure, Development, and Evolutionary Significance. Can J Zoology (2005) 83(1):122–50. doi: 10.1139/z04-163

[33] Castro A Becerra M Manso MJ Anadón R. Neuronal Organization of the Brain in the Adult Amphioxus (Branchiostoma Lanceolatum): A Study With Acetylated Tubulin Immunohistochemistry. J Comp Neurol (2015) 523(15):2211–32. doi: 10.1002/cne.23785 25846052

[34] Nozaki M Gorbman A Sower SA. Diffusion Between the Neurohypophysis and the Adenohypophysis of Lampreys, Petromyzon Marinus. Gen Comp Endocrinol (1994) 96(3):385–91. doi: 10.1006/gcen.1994.1194 7883145

[35] Gorbman A Nozaki M Kubokawa K. A Brain-Hatschek's Pit Connection in Amphioxus. Gen Comp Endocrinol (1999) 113(2):251–4. doi: 10.1006/gcen.1998.7193 10082627

[36] Gomez Mdel P Angueyra JM Nasi E. Light-Transduction in Melanopsin-Expressing Photoreceptors of Amphioxus. Proc Natl Acad Sci USA (2009) 106(22):9081–6. doi: 10.1073/pnas.0900708106 PMC2690026 19451628

[37] Hannibal J Hindersson P Knudsen SM Georg B Fahrenkrug J. The Photopigment Melanopsin Is Exclusively Present in Pituitary Adenylate Cyclase-Activating Polypeptide-Containing Retinal Ganglion Cells of the Retinohypothalamic Tract. J Neurosci (2002) 22(1):Rc191. doi: 10.1523/JNEUROSCI.22-01-j0002.2002 11756521 PMC6757615

[38] Velkeniers B Zheng L Kazemzadeh M Robberecht P Vanhaelst L Hooghe-Peters E. Effect of Pituitary Adenylate Cyclase-Activating Polypeptide 38 on Growth Hormone and Prolactin Expression. J Endocrinol (1994) 143(1):1–11. doi: 10.1677/joe.0.1430001 7964308

[39] Rousseau K Le Belle N Marchelidon J Dufour S. Evidence That Corticotropin-Releasing Hormone Acts as a Growth Hormone-Releasing Factor in a Primitive Teleost, the European Eel (Anguilla Anguilla). J Neuroendocrinol (1999) 11(5):385–92. doi: 10.1046/j.1365-2826.1999.00334.x 10320566

[40] Montero M Yon L Kikuyama S Dufour S Vaudry H. Molecular Evolution of the Growth Hormone-Releasing Hormone/Pituitary Adenylate Cyclase-Activating Polypeptide Gene Family. Functional Implication in the Regulation of Growth Hormone Secretion. J Mol Endocrinol (2000) 25(2):157–68. doi: 10.1677/jme.0.0250157 11013344

[41] Porter TE Ellestad LE Fay A Stewart JL Bossis I. Identification of the Chicken Growth Hormone-Releasing Hormone Receptor (GHRH-R) mRNA and Gene: Regulation of Anterior Pituitary GHRH-R mRNA Levels by Homologous and Heterologous Hormones. Endocrinology (2006) 147(5):2535–43. doi: 10.1210/en.2005-1534 16469800

[42] Mitchell G Sawisky GR Grey CL Wong CJ Uretsky AD Chang JP. Differential Involvement of Nitric Oxide Signaling in Dopamine and PACAP Stimulation of Growth Hormone Release in Goldfish. Gen Comp Endocrinol (2008) 155(2):318–27. doi: 10.1016/j.ygcen.2007.05.007 17574554

[43] Sahlin K Olsson R. The Wheel Organ and Hatschek's Groove in the Lancelet, Branchiostoma Lanceolatum (Cephalochordata). Acta Zoologica (1986) 67(4):201–9. doi: 10.1111/j.1463-6395.1986.tb00864.x

[44] On JS Chow BK. Molecular Evolution of Pituitary Adenylyl Cyclase-Activating Polypeptide Subfamily and Cognate Receptor Subfamily. In: Pituitary Adenylate Cyclase Activating Polypeptide—PACAP. Cham: Springer (2016). 3–17 p.

[45] Cardoso JC Vieira FA Gomes AS Power DM. The Serendipitous Origin of Chordate Secretin Peptide Family Members. BMC evolutionary Biol (2010) 10(1):1–19. doi: 10.1186/1471-2148-10-135 PMC2880984 20459630

[46] Zhang L Eiden LE. Two Ancient Neuropeptides, PACAP and AVP, Modulate Motivated Behavior at Synapses in the Extrahypothalamic Brain: A Study in Contrast. Cell Tissue Res (2019) 375(1):103–22. doi: 10.1007/s00441-018-2958-z 30519837

[47] Karpiesiuk A Palus K. Pituitary Adenylate Cyclase-Activating Polypeptide (PACAP) in Physiological and Pathological Processes Within the Gastrointestinal Tract: A Review. Int J Mol Sci (2021) 22(16):8682. doi: 10.3390/ijms22168682 34445388 PMC8395522

[48] Tjoa LT Welsch U. Electron Microscopical Observations on Kölliker's and Hatschek's Pit and on the Wheel Organ in the Head Region of Amphioxus (Branchiostoma Lanceolatum). Cell Tissue Res (1974) 153(2):175–87. doi: 10.1007/bf00226606 4442084

[49] Uchida K Murakami Y Kuraku S Hirano S Kuratani S. Development of the Adenohypophysis in the Lamprey: Evolution of Epigenetic Patterning Programs in Organogenesis. J Exp Zool B Mol Dev Evol (2003) 300(1):32–47. doi: 10.1002/jez.b.44 14984033

[50] Weng Y Song H Fang Y. Distribution of Prolactin and PRL Receptor-Like Immunoreactivities in the Nervous System, Hatschek’s Pit and Other Tissues of Amphioxus Branchiostoma Belcheri. Acta Zool Sin (2006) 52:907–15.

[51] Nozaki M Gorbman A. The Question of Functional Homology of Hatschek's Pit of Amphioxus (Branchiostoma Belcheri) and the Vertebrate Adenohypophysis. Zoological Sci (1992) 9(2):387–95.

[52] Candiani S Holland ND Oliveri D Parodi M Pestarino M. Expression of the Amphioxus Pit-1 Gene (AmphiPOU1F1/Pit-1) Exclusively in the Developing Preoral Organ, a Putative Homolog of the Vertebrate Adenohypophysis. Brain Res Bull (2008) 75(2-4):324–30. doi: 10.1016/j.brainresbull.2007.10.023 18331893

[53] Tam JK Lee LT Chow BK. PACAP-Related Peptide (PRP)—molecular Evolution and Potential Functions. Peptides (2007) 28(9):1920–9. doi: 10.1016/j.peptides.2007.07.011 17714829

[54] Parker DB Power ME Swanson P Rivier J Sherwood NM. Exon Skipping in the Gene Encoding Pituitary Adenylate Cyclase-Activating Polypeptide in Salmon Alters the Expression of Two Hormones That Stimulate Growth Hormone Release. Endocrinology (1997) 138(1):414–23. doi: 10.1210/endo.138.1.4830 8977431

[55] Montero M Yon L Rousseau K Arimura A Fournier A Dufour S. Distribution, Characterization, and Growth Hormone-Releasing Activity of Pituitary Adenylate Cyclase-Activating Polypeptide in the European Eel, Anguilla Anguilla. Endocrinology (1998) 139(10):4300–10. doi: 10.1210/endo.139.10.6239 9751513

