# Supplementary material for: PACAP/GCGa Is an Important Modulator of the Amphioxus CNS-Hatschek’s Pit Axis, the Homolog of the Vertebrate Hypothalamic-Pituitary Axis in the Basal Chordates
Source: Front Endocrinol (Lausanne). 2022 Apr 14;13:850040. doi: 10.3389/fendo.2022.850040 (PMC9049855; doi:10.3389/fendo.2022.850040)
Supplement: Supplementary file 1 [file DataSheet_1.pdf]

Supplementary Data

TTACTCATTCGAACCTGTGGATCCACACTAGAGAGTAGTTGAGGACTTAACCGGGTGGCGGTCTGCGCACGCTAACATT
GTCCGTGCTCCCGTACCAACGCCAGGTGAGACGTCAGGTGAAGAAGAGGACGAGCAACAAC

```
ATGCGTTGCGGACTGAGCTGGCTGCTGTTCCTGCTGACGTACACGTTAGTTCTGCTGGCC
 M  R  C  G  L  S  W  L  L  F  L  L  T  Y  T  L  V  L  L  A
GTGACAGCCTTACCGACGGGCGACAGCACACTGGCACGCGTGAGAGAGAGGCGGCAGCTG
 V  T  A  L  P  T  G  D  S  T  L  A  R  V  R  E  R  R  Q  L
GGAGACCAGTCGATCACCAGCGAGATGTCCGTCAGACTACGGGAGGCCGAGGCCCGCCGG
 G  D  Q  S  I  T  S  E  M  S  V  R  L  R  E  A  E  A  R  R
CTGCTGCAAAGCCTCATGGCGAAACAGGGTAAAAGAGCGATGACGGAAGGTGACATGTTC
 L  L  Q  S  L  M  A  K  Q  G  K  R  A  M  T  E  G  D  M  F
AGTAGCCAGTCCCAGCAGAAGTGGAGGCGCGACCCTGTGGCCGAGTTTCTGGACCAGCTG
 S  S  Q  S  Q  Q  K  W  R  R  D  P  V  A  E  F  L  D  Q  L
TACGAACAGGAGGACTTCTAA
 Y  E  Q  E  D  F  *
```

CATCGGCACGTCCACACTTTGTAAGGCTACTTTGCCGTACCTGAAGCAAACGAGGACCCAGCTCAGGACATCCGATGGA
CTAACAGTTTGACCTGTCGGGTCAGAGGTCACGGACGGGTCGTCTACTTGCATGCC

Supplementary Figure 1 | Full-length cDNA of the *PACAP/GCGa* peptide precursor isolated from *B. belcheri*. Amino sequences labeled with yellow correspond to the predicted signal peptide (http://www.cbs.dtu.dk/services/SignalP/) and in red represent the predicted PACAP/GCGa mature peptide according to Mirabeau and Joly (1). The 5' or 3' UTR sequences are in italics and the asterisk "*" represents the STOP codon.

TTACTCATTCGAACCTGTGGATCCACACTAGAGAGTAGTTGAGGACTTAACCGGGTGGCGGTCTGCGCACGCTAACATT
GTCCGTGCTCCCGTACCGTAACGCCAGGTGAGGCGTCAGGTGAAGAAGAGGACGAGCAACAAC

ATGCGTTGCGGACTGAGCTGGCTGCTGTTCCTGCTGACGTACACGTTAGTTCTGCTGGCC
M R C G L S W L L F L L T Y T L V L L A
GTGACAGCCTTACCGACGGGCGACAGCACACTGGCACGGGGCCGCAGGGCGCTGGGTGAC
V T A L P T G D S T L A R G R R A L G D
CAAGGGTTCACCAGTGACCTCGCGAGCAAGCTGAGCGAAGCCGAGGCCAGAAGGATGATT
Q G F T S D L A S K L S E A E A R R M I
CAGAACCTCATGGCTCAGGCCATCGGCAAGCGCTTCAGTCCAACTGACGGGCAGGAGCAG
Q N L M A Q A I G K R F S P T D G Q E Q
CTGCAGGCCTCCAAGCGCCAGCTGGGCGACCAGGGCGTCACTAGCGCGCTGGCCGCCCGG
L Q A S K R Q L G D Q G V T S A L A A R
CTCGAGCAGGCCGAGGCCCGCCAGTACATCAAGGACCTCCTGGAGCAGGCTGTCGGGAAG
L E Q A E A R Q Y I K D L L E Q A V G K
CGATCAGTGGCCAAGCGCTCCACTGCAGACGCACCAGCTAGCAGGAAGCGACGGGCTTTA
R S V A K R S T A D A P A S R K R R A L
GCCGACCAGGGGCCGGAGGACCAGCCGCCTGCTGAGGCCCGTAAGATTTTGATGACCCTC
A D Q G P E D Q P P A E A R K I L M T L
CTGCCGATCAGAGCGATGACGGAAGGTGACATGTTCAGTAGCCAGTCCCAGCAGAAGTGG
L P I R A M T E G D M F S S Q S Q Q K W
AGGCGCGACCCTGTGGCCGAGTTTCTGGACCAGCTGTACGAACAGGAGGACTTCTAA
R R D P V A E F L D Q L Y E Q E D F *

CATCGGCACGTCCACACTTTGTAAGGCTACTTTGCCGTACCTGAAGCAAACGAGGACCCAGCTCAGGACATCCGATGGA
CTAACAGTTTGACCTGTCGGGTCAGAGGTCACGGACGGGTCGTCTACTTGCATGCC

Supplementary Figure 2 | Full-length cDNA of the *PACAP/GCGb* and *c* peptide precursor isolated from *B. belcheri.* Amino acid sequences labeled in yellow correspond to the predicted signal peptide (http://www.cbs.dtu.dk/services/SignalP/) and in red and green respectively represent the PACAP/GCGb and c mature peptide according to Mirabeau and Joly (1). The 5’ or 3’ UTR sequences are in italics and the asterisk “*” represents the STOP codon.

```
ATGCGCTGCGGACTGAGCTGGCTGCTGTTCTTCCTGACGTACACGTTAGTTCTACTGGCC
 M  R  C  G  L  S  W  L  L  F  F  L  T  Y  T  L  V  L  L  A

GTGACAGCCTTACCGACGGGCGACTCCACATTGGCACGAGTGAGAGAGAAGCGGCAGCTT
 V  T  A  L  P  T  G  D  S  T  L  A  R  V  R  E  K  R  Q  L

GGAGACCAGTCCATCACCAGCGAGATGTCCGTCAGACTCAGGGAGGCCGAGGCCCGCCGG
 G  D  Q  S  I  T  S  E  M  S  V  R  L  R  E  A  E  A  R  R

CTGCTCCAAAGCCTCATGGCTAAACAGGGTAAAAGAGCGGTGACCGAAGAGGACATGTAC
 L  L  Q  S  L  M  A  K  Q  G  K  R  A  V  T  E  E  D  M  Y

AGCCAGTCCCAGCAGAAGTGGAGGCGCGACCCTGTCGCCGAGTTCCTGGACCGACTGTAC
 S  Q  S  Q  Q  K  W  R  R  D  P  V  A  E  F  L  D  R  L  Y

GAGCAGGGGGACTTCTAA
 E  Q  G  D  F  *
```

Supplementary Figure 3 | Sequence of *B. floridae* PACAP/GCGa peptide precursor coding domain sequence. Amino sequences labeled in yellow correspond to the predicted signal peptide (http://www.cbs.dtu.dk/services/SignalP/) and in red represent the PACAP/GCGa mature peptide according to Mirabeau and Joly (1). The asterisk "*" represents the STOP codon.

```
ATGCGCTGCGGACTGAGCTGGCTGCTGTTCTTCCTGACGTACACGTTAGTTCTACTGGCC
 M  R  C  G  L  S  W  L  L  F  F  L  T  Y  T  L  V  L  L  A

GTGACAGCCTTACCGACGGGCGACTCCACATTGGCACGGGATCGCAGGGCTCTGGGTGAC
 V  T  A  L  P  T  G  D  S  T  L  A  R  D  R  R  A  L  G  D

CAGGGGTTTACCAGTGACCTTGCTAGCAAGCTGAGCGAAGCCGAGGCCAGGAGGATGATC
 Q  G  F  T  S  D  L  A  S  K  L  S  E  A  E  A  R  R  M  I

CAGACCCTCATGGCTCAGGCCATCGGTAAGAGGTTCAGTCCGGCTGAACAGCAGGAACCC
 Q  T  L  M  A  Q  A  I  G  K  R  F  S  P  A  E  Q  Q  E  P

CTGGAAGCCTCCAAGCGCCAGCTGGGCGACCAGGGGGTCACTAGCGCGCTGGCCGCCCGA
 L  E  A  S  K  R  Q  L  G  D  Q  G  V  T  S  A  L  A  A  R

CTTGAACAGGCGGAGGCCCGTCAGTACATCAAGGACCTCCTGGCTCAGGCTGTCGGGAAG
 L  E  Q  A  E  A  R  Q  Y  I  K  D  L  L  A  Q  A  V  G  K

CGATCGGGCGGGGTGGCCAAGCGCTCCACAGCGGAGGGAGCGGCTAGCAGGAAGAGACGA
 R  S  G  G  V  A  K  R  S  T  A  E  G  A  A  S  R  K  R  R

GCTTTGACAGACCAGGGGCCGATGGACCATCCGCCTGTTGGACAGGAGGCGTCCGAGGCC
 A  L  T  D  Q  G  P  M  D  H  P  P  V  G  Q  E  A  S  E  A

CGTAAGATTTTGATGACCCTCCTGTTCGGCTACGGGAAGACCAGAGCGATGACCGAAGAG
 R  K  I  L  M  T  L  L  F  G  Y  G  K  T  R  A  M  T  E  E

GACATGTACAGCCAGTCCCAGCAGAAGTGGAGGCGCGACCCTGTCGCCGAGTTCCTGGAC
 D  M  Y  S  Q  S  Q  Q  K  W  R  R  D  P  V  A  E  F  L  D

CGACTGTACAAGCAGGAGGACTTCTAA
 R  L  Y  K  Q  E  D  F  *
```

Supplementary Figure 4 | Sequence of the *B floridae* PACAP/GCGb and c peptide precursor coding domain sequence. Amino acid sequences labeled in yellow correspond to the predicted signal peptide (http://www.cbs.dtu.dk/services/SignalP/) and in red and green respectively represent the PACAP/GCGb and c mature peptide according to Mirabeau and Joly (1). The asterisk "*" represents the STOP codon.

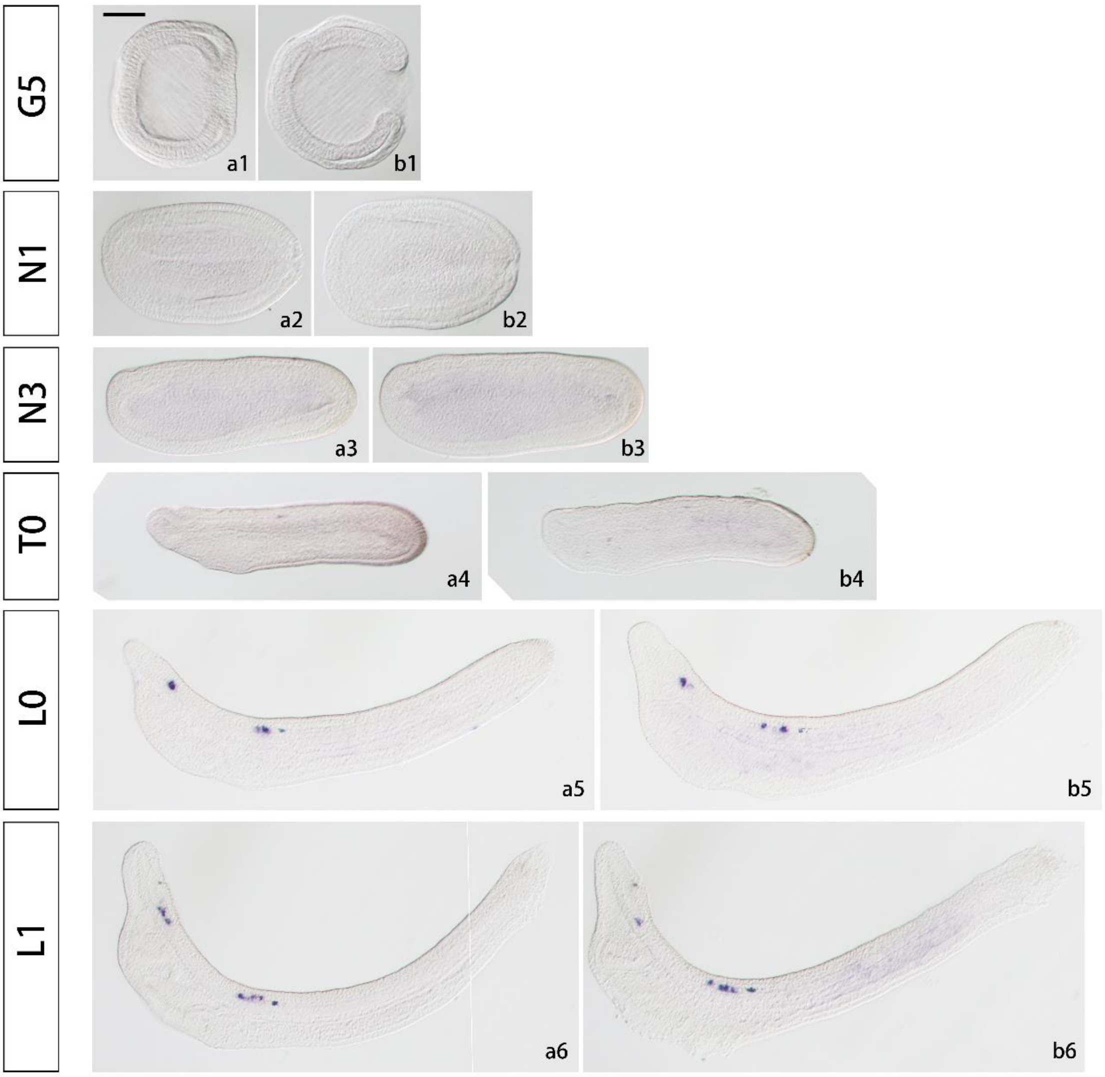

Supplementary Figure 5 | Expression profile of *PACAP/GCGa* (a1-6) and *PACAP/GCGbc* (b1-6) peptide precursors in various early developmental stages of *B. floridae*. The amphioxus developmental stages are according to recently updated staging system (2). All images share the same scale bar (100 µm). Image **(a5)**, **(b5)** and **(b6)** were merged using Photoshop software, while image **(a6)** are spliced artificially from two images.

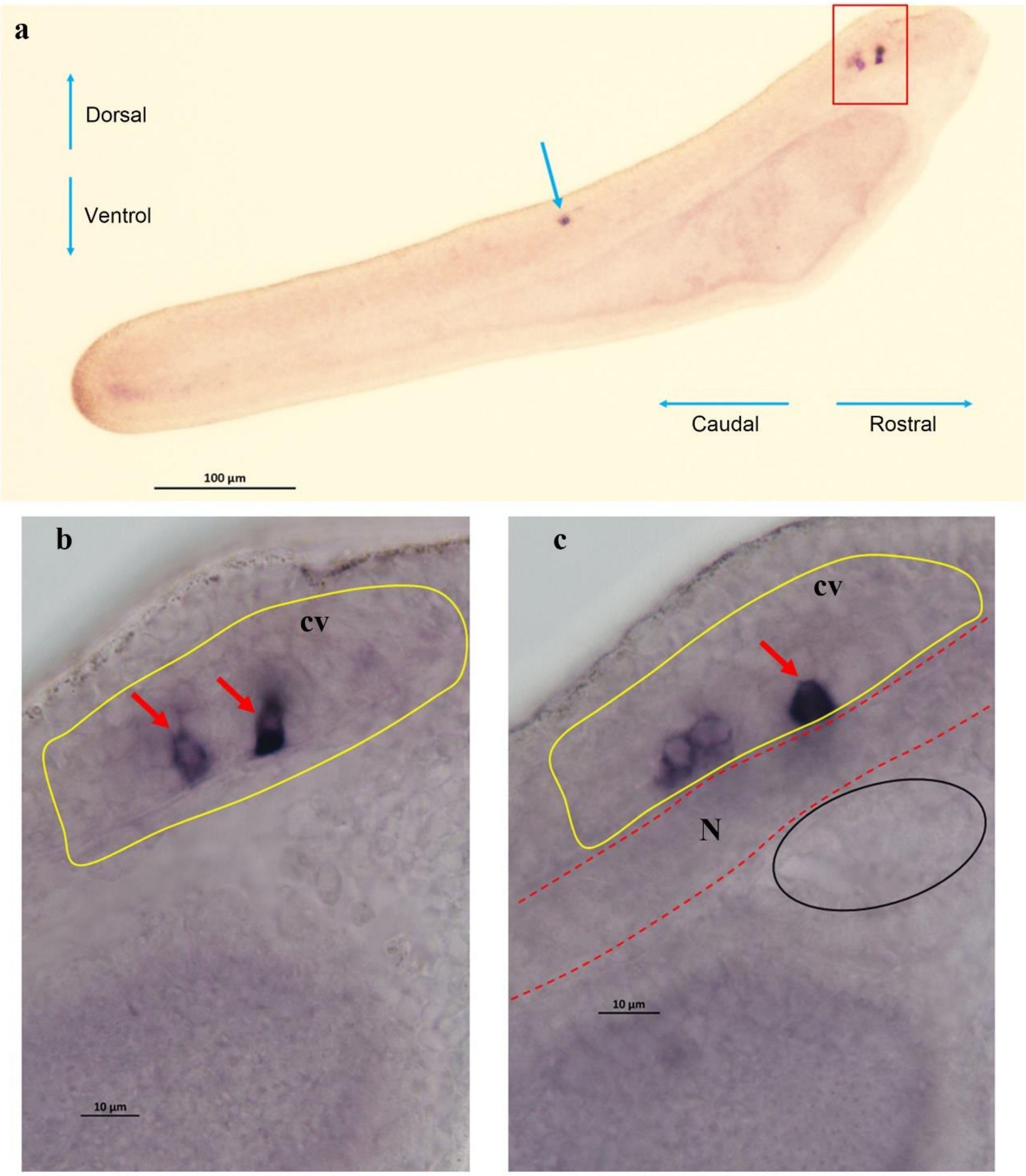

Supplementary Figure 6 | Expression of *PACAP/GCGa* peptide precursors during T0 stage in *B. floridae*. (A) Right side body view at T0 stage. **(B)** and **(C)** are higher magnification images of the positive cells limited by the red box in **(A)**. The yellow lines demarcated the cerebral vesicle. The red dotted lines demarcated the notochord region. The black oval shape demarcates the left diverticulum. The first pigment spot is marked by a blue arrow and the *PACAP/GCGa* positive cells are indicated by a red arrow. cv, cerebral vesicle; N, notochord. The scale bars are indicated in the images.

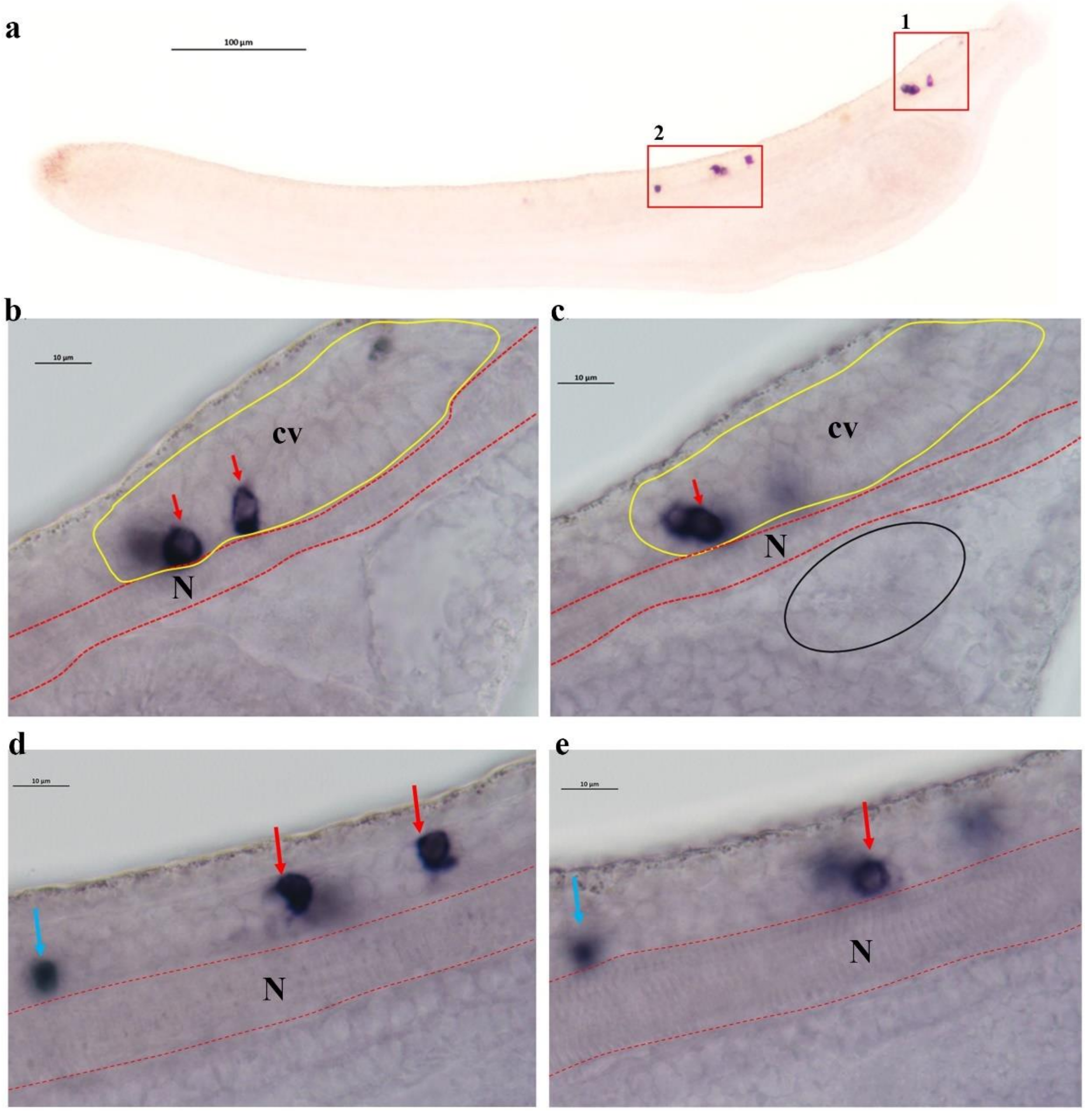

Supplementary Figure 7 | Expression of *PACAP/GCGa* peptide precursor at L0 stage in *B. floridae*. (A) Right side view of the L0 stage. The red boxed regions 1 and 2 where positive cells were identified are shown in higher magnification in **(B, C)** and **(D, E)**, respectively. **(B)** Staining of the topmost layer. Two cells were stained in this layer and located in the posterior region of the cerebral vesicle. **(C)** A lower layer of cells in which a *PACAP/GCGa* positive cell was identified and located in the posterior region of the cerebral vesicle. **(D)** Topmost layer of box 2 where positive cells were identified. **(E)** Lower layer of box 2 where a positive cell was stained. The yellow lines demarcated the cerebral vesicle and the red dotted lines demarcated the notochord region. The black oval shape line demarcates the left diverticulum. The first pigment spot is

indicated by a blue arrow and the *PACAP/GCGa* positive cells were pointed out by the red arrow. cv, cerebral vesicle; N, notochord. The scale bars are indicated in the images.

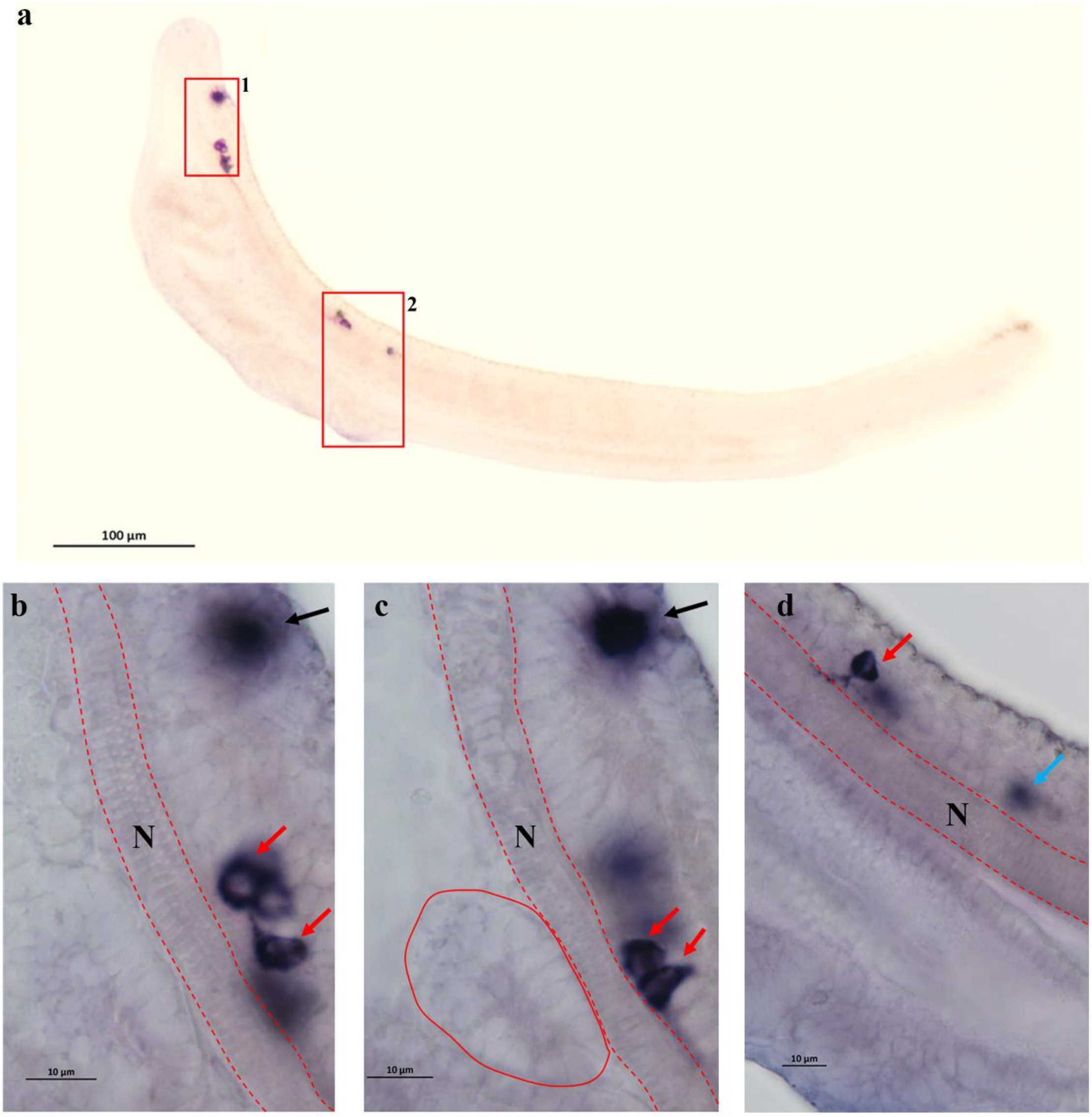

Supplementary Figure 8 | Expression of *PACAP/GCGa* peptide precursor at L1 stage in *B. floridae*. (A) Left side view at L1 stage. The red boxed 1 and 2 indicate the regions where positive cells were identified and **(B, C)** and **(D)**, represent their higher magnification images, respectively. The blue arrow indicates the first pigment spot. **(B)**, The topmost layer where staining is observed. **(C)** A lower layer of cells compared to image **b** where positive cells were identified. **(D)** The topmost layer of box 2 where positive cells was identified. Red dotted lines demarcated the notochord region. The red circle demarcated the pre-oral pit. The first pigment spot is marked by a blue arrow, the black arrow points out the frontal eye pigment and the

PACAP/GCGa positive cells were pointed out by red arrow. N, notochord. The scale bars are indicated in the images.

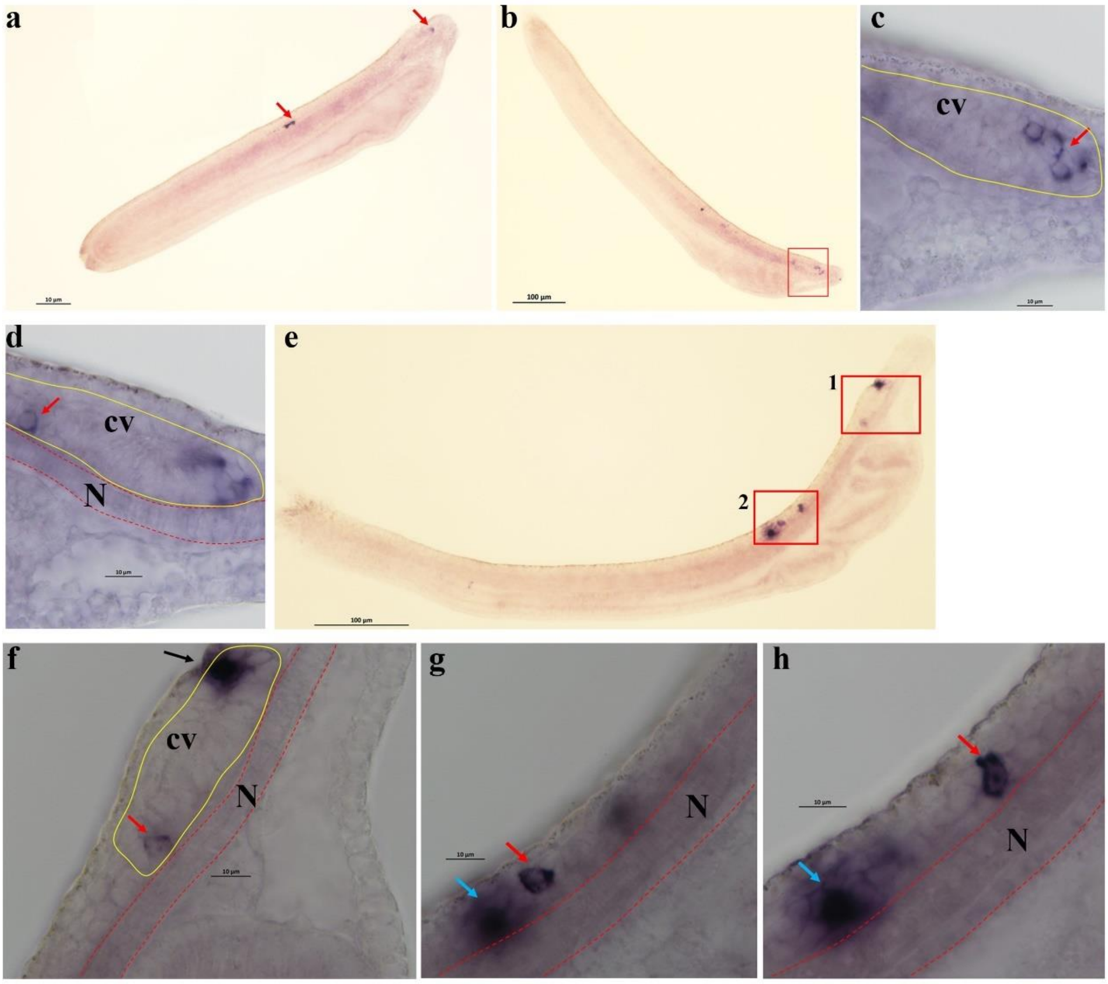

Supplementary Figure 9 | Expression of *PACAP/GCGbc* peptide precursor at T0, L0 and L2 stages in *B. floridae*. **(A)** Image of the right side view at T0 stage. **(B)** Image of the right side view at L0 stage. Positive cells were detected in the anterior and posterior cerebral vesicle and the observation is clearer at the magnified images **(C)** and **(D)**. **(E)** Image of the right side view at L2 stage. Boxed regions 1 and 2 are detailed in **(F)** and **(G)-(H)**, respectively. **(F)** A single positive cell signal was observed in the posterior cerebral vesicle. **(G)-(H)** In the successive layer, two positive cells were also identified. Yellow lines demarcated basically the cerebral vesicle. Red dotted lines demarcated the notochord region. The first pigment spot is marked by a blue arrow, the black arrow points out the frontal eye pigment and the *PACAP/GCGbc* positive cells were pointed out by red arrows. cv, cerebral vesicle; N, notochord. The scale

bars are indicated in the images.

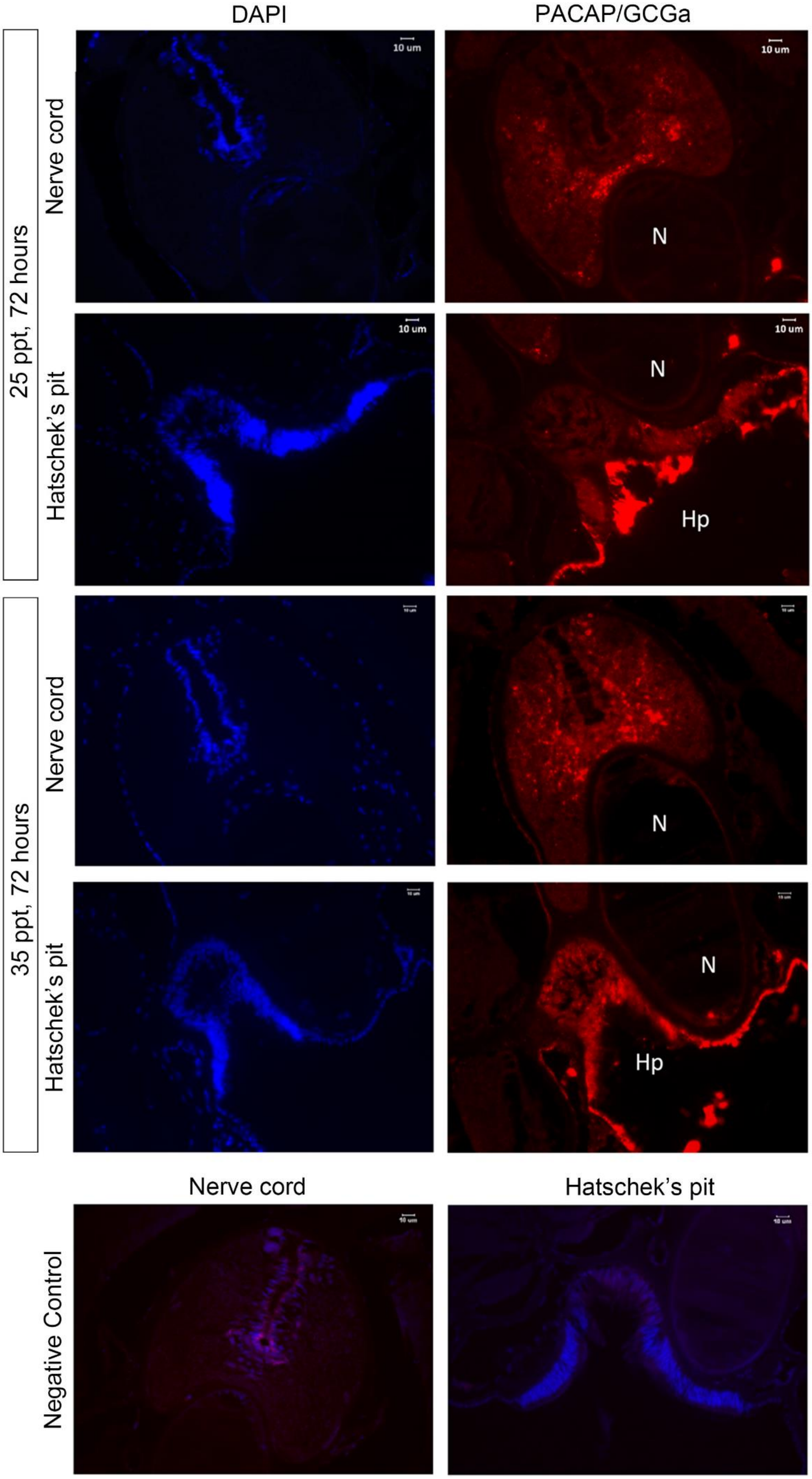

DAPI
PACAP/GCGa
25 ppt, 72 hours
Nerve cord
Hatschek's pit
35 ppt, 72 hours
Nerve cord
Hatschek's pit
N
Hp
10 um
Negative Control
Nerve cord
Hatschek's pit

Supplementary Figure 10 | Effect of change in ambient salinity on the PACAP/GCGa level in the Hatschek's pit and nerve cord by immunofluorescence staining. Immunofluorescence digital images of DAPI staining (blue), PACAP/GCGa (red) positive signals and negative control (merged). Negative controls (merged) represent the result of incubations in which primary antibody with overnight incubation with antigen in 1:10 molar ratio was used to replace primary antibody alone. Amphioxus that lived in 25‰ for 72 hours are used for control group, while that lived in 35‰ for 72 hours are used for treated group. N: notochord; Hp: Hatschek's pit. The scale bar is 10 μm and is indicated in the images.

Supplementary Table S1. List of the primer sequences used for cloning the BbPACAP/GCGs cDNA and BfPACAP/GCGs.

	Amplification	Primer	Sequence (5’→3’)
BbPACAP/GCGs	5’ RACE	bbP+G -5RR1	ATCTCGCTGGTGATCGACTG
		bbP+G -5RR2	CGTGCCAGTGTGCTGTCGCC
	3’RACE	bbP+G -3RF1	TCCGCTGCAGACGCACCAGC
		bbP+G -3RF2	CCCTCCTGCCGATCAGAGCG
	Full length clone	bbP+G -FLF1	ATAGAATCTTGGGTTGGTTC
		bbP+G -FLR1	GCTCATGTTTACATTGTATC
		bbP+G -FLF2	TTACTCATTCGAACCTGTGG
		bbP+G -FLR2	CGGCATGCAAGTAGACGACC
BfPACAP/GCGs		Fw	ATGCGCTGCGGACTGAGCTG
		Rv	TTAGAAGTCCCCCTGCTCGT

Supplementary Table S2. List of the primer sequence used for RT-qPCR.

	Accession number	Sequences (5’→ 3’)	product size (bp)
ef1 α	Bb_188770F	Fw: GATTGTGGCTGCTGGTACTG<br>Rv: GGTTGTAGCCGATCTTCTTG	211
PACAP/GCGR	Bb_320400R	Fw: ACTTCATCGCTGCCAACTAC<br>Rv: AGATCCACCAGTACTGCAGG	219
PACAP/GCGa	Bb_267100R	Fw: AGACCAGTCGATCACCAGCG<br>Rv: GCCGATGTTAGAAGTCCTCC	206
GHl	Bb_070560F	Fw: CGCTAGAGTGGAGAAACTTG<br>Rv: AGGACGTGGTTCGCTACAGC	201

Supplementary Table S3. List of the primer sequence used for synthesizing the mRNA probes for in situ hybridization.

	Sequences (5'→ 3')
BbGHl (Bb_070560F)	Fw: CGTCGCCGCTAGACTGTACC<br>Rv: TGTATTTGCGGCTCAGTAGG
BfPACAP/GCGa*<br>(Supplementary Figure 5)	Fw: AGTGAGAGAGAAGCGGCAG<br>Rv: CGGTCACCGCTCTTTTACCC
BfPACAP/GCGbc	Fw: AGTGAGAGAGAAGCGGCAG<br>Rv: CGGTCACCGCTCTTTTACCC
BfPACAP/GCGa* (Supplementary Figure 6-8)	Fw: GGTAAGGACATTCGGACCTG<br>Rv: CCTGGTACAGCCAAGTGGC
BfPACAP/GCGa*<br>(Supplementary Figure 9)	Fw: AGTGACCTTGCTAGCAAGC<br>Rv: ACTGGCTGTACATGTCCTC

*- Different primers were used to produce the probes

Supplementary Table S4. Mature peptide sequences of the synthetic PACAP/GCG peptides produced.

Peptide name	Amino acid sequence	References
BfPACAP/GCGa	QLGDQSITSEMSVRLREAEARRLLQSLMAKQ	(Mirabeau and Joly 2013)
BfPACAP/GCGb	ALGDQGFTSDLASKLSEAEARRMIQTLMAQAI	(Mirabeau and Joly 2013)
BfPACAP/GCGc	QLGDQGVTSALAARLEQAEARQYIKDLLAQAV	(Mirabeau and Joly 2013)

Supplementary Table S5. Amino acid sequences used for raising the specific antibodies.

	Antigen sequence used for immunization
PACAP/GCGa	CMAKQGKRAMTEGDM
GHl	CTDVTEIMSTSLRDP

Note: The cysteine underlined is the extra amino acid added to help conjugation.

REFERENCES

1. O. Mirabeau and J. S. Joly. Molecular evolution of peptidergic signaling systems in bilaterians. *Proc Natl Acad Sci U S A* (2013) 110(22):E2028-37. doi: 10.1073/pnas.1219956110

2. J. E. Carvalho, F. Lahaye, L. W. Yong, J. C. Croce, H. Escriva, J. K. Yu, et al. An Updated Staging System for Cephalochordate Development: One Table Suits Them All. *Frontiers in Cell and Developmental Biology* (2021) 9. doi: 10.3389/fcell.2021.668006
